# The Mitochondrial Unfoldase-Peptidase Complex ClpXP Controls Bioenergetics Stress and Metastasis

**DOI:** 10.1371/journal.pbio.1002507

**Published:** 2016-07-07

**Authors:** Jae Ho Seo, Dayana B. Rivadeneira, M. Cecilia Caino, Young Chan Chae, David W. Speicher, Hsin-Yao Tang, Valentina Vaira, Silvano Bosari, Alessandro Palleschi, Paolo Rampini, Andrew V. Kossenkov, Lucia R. Languino, Dario C. Altieri

**Affiliations:** 1 Prostate Cancer Discovery and Development Program, The Wistar Institute, Philadelphia, Pennsylvania, United States of America; 2 Tumor Microenvironment and Metastasis Program, The Wistar Institute, Philadelphia, Pennsylvania, United States of America; 3 Molecular and Cellular Oncogenesis Program, The Wistar Institute, Philadelphia, Pennsylvania, United States of America; 4 Center for Systems and Computational Biology, The Wistar Institute, Philadelphia, Pennsylvania, United States of America; 5 Division of Pathology, Fondazione IRCCS Ca’ Granda Ospedale Maggiore Policlinico, Milan, Italy; 6 Department of Pathophysiology and Transplantation, University of Milan, Milan, Italy; 7 Division of Thoracic Surgery, Fondazione IRCCS Ca’ Granda Ospedale Maggiore Policlinico, Milan, Italy; 8 Division of Neurosurgery, Fondazione IRCCS Ca’ Granda Ospedale Maggiore Policlinico, Milan, Italy; 9 Department of Cancer Biology and Kimmel Cancer Center, Thomas Jefferson University, Philadelphia, Pennsylvania, United States of America; University of California Los Angeles, UNITED STATES

## Abstract

Mitochondria must buffer the risk of proteotoxic stress to preserve bioenergetics, but the role of these mechanisms in disease is poorly understood. Using a proteomics screen, we now show that the mitochondrial unfoldase-peptidase complex ClpXP associates with the oncoprotein survivin and the respiratory chain Complex II subunit succinate dehydrogenase B (SDHB) in mitochondria of tumor cells. Knockdown of ClpXP subunits ClpP or ClpX induces the accumulation of misfolded SDHB, impairing oxidative phosphorylation and ATP production while activating “stress” signals of 5′ adenosine monophosphate-activated protein kinase (AMPK) phosphorylation and autophagy. Deregulated mitochondrial respiration induced by ClpXP targeting causes oxidative stress, which in turn reduces tumor cell proliferation, suppresses cell motility, and abolishes metastatic dissemination in vivo. ClpP is universally overexpressed in primary and metastatic human cancer, correlating with shortened patient survival. Therefore, tumors exploit ClpXP-directed proteostasis to maintain mitochondrial bioenergetics, buffer oxidative stress, and enable metastatic competence. This pathway may provide a “drugable” therapeutic target in cancer.

## Abstract 2

Exploitation of mitochondrial protein folding quality control via the mitochondrial unfoldase-peptidase complex ClpXP is critical for tumor cell proliferation, invasion, and metastatic competence, and represents a potential therapeutic target.

## Author Summary

As the powerhouse of the cell and a pivotal hub for oxidative stress, mitochondria must tightly control the state of the proteins that they contain, quickly eliminating misfolded, aggregated, or otherwise damaged proteins. Here, we show that tumor mitochondria manage their set of proteins by assembling an integrated network of protein homeostasis, or proteostasis, that controls both the folding and degradation of proteins. This protein complex is formed by the unfoldase-peptidase ClpXP, survivin, and the Hsp90-like chaperone TRAP-1, and regulates the function of the oxidative phosphorylation Complex II subunit succinate dehydrogenase B (SDHB). We find that interference with this process impairs energy production, promotes oxidative stress, and shuts down critical downstream signals important for tumor cell proliferation, invasion, and metastatic dissemination in vivo. Our results suggest that the mitochondrial proteostasis network may offer therapeutic opportunities in advanced disease.

## Introduction

The control of protein homeostasis, or proteostasis, occupies a central, evolutionary-conserved role in organismal integrity and flexible adaptation to environmental “stress” [1]. This pathway involves mechanisms of chaperone-directed protein (re)folding [2] as well as removal of aggregated or misfolded proteins via proteolytic degradation [3]. Defects in either process impair organelle function, in particular the endoplasmic reticulum (ER) [4] and mitochondria [5], activating an unfolded protein response (UPR) that may culminate in cell death and tissue damage [6].

There is also evidence that a heightened proteostatic threshold can contribute to disease, in particular cancer, by buffering the risk of proteotoxic stress associated with the biosynthetic needs of transformed cells. Accordingly, molecular chaperones of the heat shock protein-90 (Hsp90) family, including Hsp90 [7] and its homolog, TNFR-associated molecule-1 (TRAP-1) [8], become overexpressed in mitochondria of tumor cells compared to normal tissues [9] and preserve the folding and activity of key effectors of organelle homeostasis [10]. In turn, the heightened proteostatic environment prevents the emergence of a mitochondrial UPR [11], antagonizes cyclophilin D-dependent apoptosis [9], and maintains bioenergetics [10], including oxidative phosphorylation [12], correlating with unfavorable disease outcome in cancer patients [13].

What has remained unclear, however, is whether chaperone-directed protein folding is the sole mechanism of mitochondrial proteostasis in cancer [10]. In this context, mitochondria contain an evolutionary-conserved, ATP-dependent unfoldase-peptidase protein complex, ClpXP [14], which mediates proteolytic removal of misfolded proteins [15]. There is evidence that this pathway may regulate a mitochondrial UPR [16,17] and contribute to human disease pathogenesis [18].

In this study, we investigated mechanisms of mitochondrial proteostasis as a potential driver of tumor progression.

## Results

### Identification of ClpXP as a Novel Survivin-Associated Molecule

**Fig 1:**
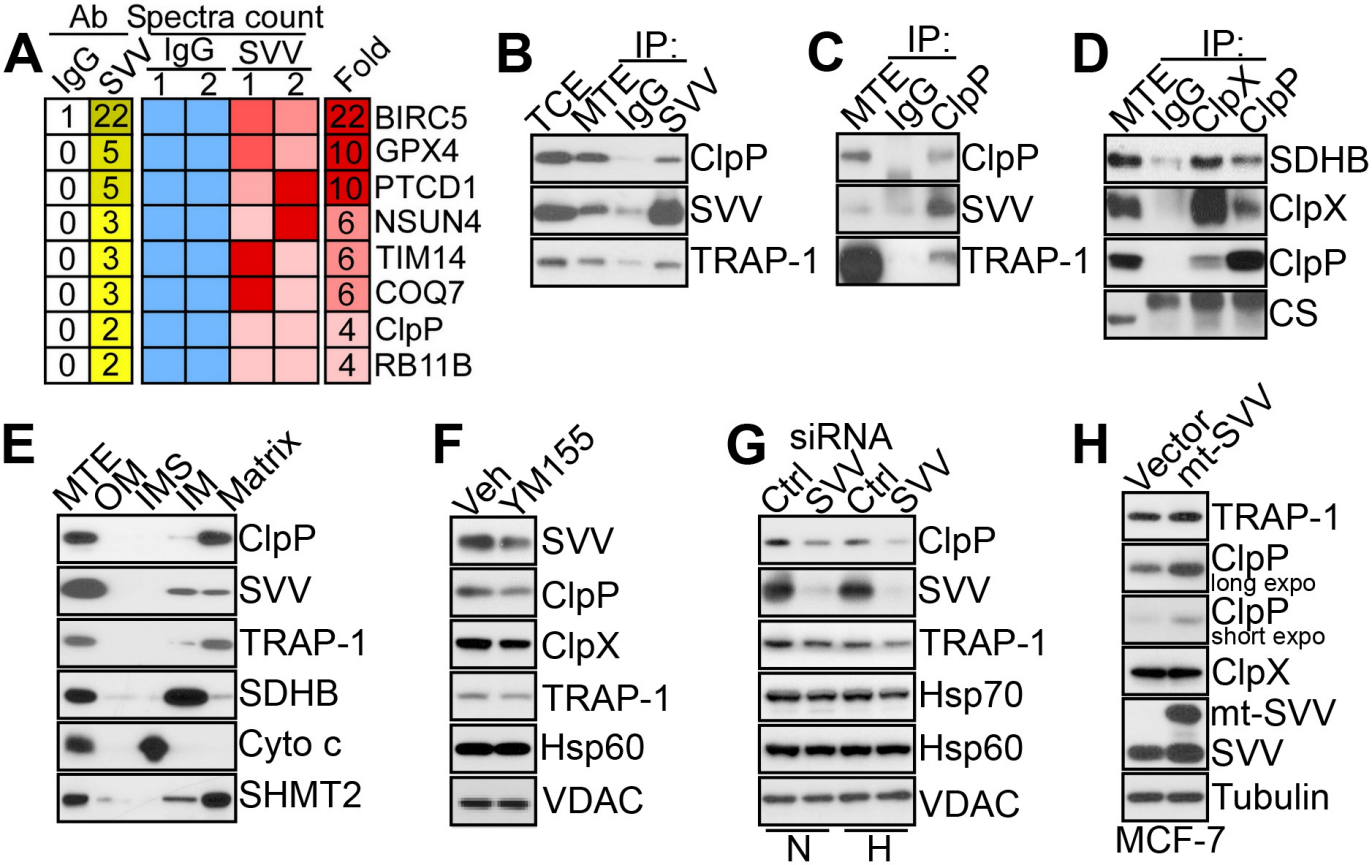
Survivin-ClpXP interaction. (**A**) Heatmap of proteomics identification of mitochondrial proteins co-precipitated with an antibody to survivin (SVV) or control IgG from PC3 mitochondrial extracts. Two independent replicates per condition are shown. A fold enrichment in spectra counts (SVV/IgG) is shown. (**B–D**) Mitochondrial extracts from PC3 cells were immunoprecipitated (IP) with antibodies to survivin (SVV) (**B**), ClpP (**C**), ClpP or ClpX (**D**), or non-binding IgG and analyzed by western blotting. TCE, total cell extracts. MTE, mitochondrial extracts. CS, citrate synthase. (**E**) Submitochondrial fractions representative of outer membrane (OM), inter-membrane space (IMS), inner membrane (IM), or matrix were isolated from PC3 cells and analyzed by western blotting. (**F**) PC3 cells were treated with vehicle (Veh) or small molecule survivin (SVV) suppressant YM155 and analyzed by western blotting. (**G**) PC3 cells in normoxia (N) or hypoxia (H) were transfected with control non-targeting siRNA (Ctrl) or survivin (SVV)-directed siRNA and analyzed by western blotting. (**H**) MCF-7 cells were transfected with vector or mitochondrial-targeted (mt) survivin (SVV) cDNA and analyzed by western blotting. Expo, exposure.

Previous studies have shown that a pool of the inhibitor-of-apoptosis (IAP) protein survivin [19] localizes to mitochondria and contributes to the stability of oxidative phosphorylation Complex II subunit succinate dehydrogenase B (SDHB) [20]. To further characterize this pathway, we carried out a proteomics screen for additional survivin-associated molecules in mitochondria, using prostate adenocarcinoma PC3 cells as a tumor model (S1 Methods). In this screen, molecules associated with mitochondrial survivin comprised regulators of organelle trafficking (Rab11), assembly of respiratory chain complexes (PTCD1), oxidative stress (GPX4), mitoribosomal transferase activity (NSUN4), ubiquinone biosynthesis (COQ7), and the AAA+ peptidase subunit of the ClpXP complex, ClpP (Fig 1A).

Consistent with these results, survivin immune complexes precipitated from mitochondria of PC3 cells contained ClpP as well as TRAP-1 (Fig 1B) [20]. Reciprocally, ClpP co-immunoprecipitated with survivin and TRAP-1 in tumor mitochondria (Fig 1C). In addition, ClpX and ClpP mutually associated with each other as well as with SDHB in co-immunoprecipitation experiments (Fig 1D), consistent with the assembly of a survivin-ClpXP [14]-SDHB complex in tumor mitochondria. When analyzed for submitochondrial distribution, survivin, ClpP, and TRAP-1 co-localized within the organelle matrix in PC3 cells (Fig 1E). In addition, a fraction of TRAP-1 and survivin localized to the inner mitochondrial membrane (Fig 1E), in agreement with recent observations [20].

To begin investigating the role of a survivin-ClpXP complex in mitochondria, we next exposed PC3 cells to YM155, a small molecule survivin suppressant currently examined in the clinic as an anticancer drug. In these experiments, YM155 treatment was associated with reduced expression of ClpP (Fig 1F). As an independent approach, we next silenced survivin using our validated small interfering RNA (siRNA) sequences [20]. Survivin knockdown significantly reduced the levels of ClpP in mitochondria of PC3 cells, especially under conditions of hypoxic stress (Fig 1G). In contrast, YM155 treatment (Fig 1F) or siRNA silencing of survivin (Fig 1G) did not affect the expression of mitochondrial chaperones Hsp70 or Hsp60, or the voltage-dependent anion channel (VDAC). Next, we used cycloheximide block experiments to quantify changes in ClpXP stability after survivin targeting. In these experiments, YM155 treatment (S1A and S1B Fig) or siRNA silencing of survivin (S1C and S1D Fig) resulted in accelerated turnover of ClpP, compared to control siRNA transfectants. This regulation occurred at the protein level because survivin knockdown did not affect ClpP mRNA expression (S1E Fig). In reciprocal experiments, transfection of PC3 cells with a survivin variant engineered to selectively accumulate in mitochondria [20] increased ClpP levels in mitochondria (Fig 1H). In contrast, targeting survivin with YM155 (Fig 1F) or siRNA knockdown (S1C and S1D Fig) or, conversely, over-expression of mitochondrial survivin (Fig 1H) did not appreciably affect ClpX levels. This suggests that ClpP is the primary binding partner of survivin in the ClpXP complex, consistent with the results of the proteomics screen (Fig 1A).

### ClpXP Regulation of Mitochondrial Complex II Folding and Activity

**Fig 2:**
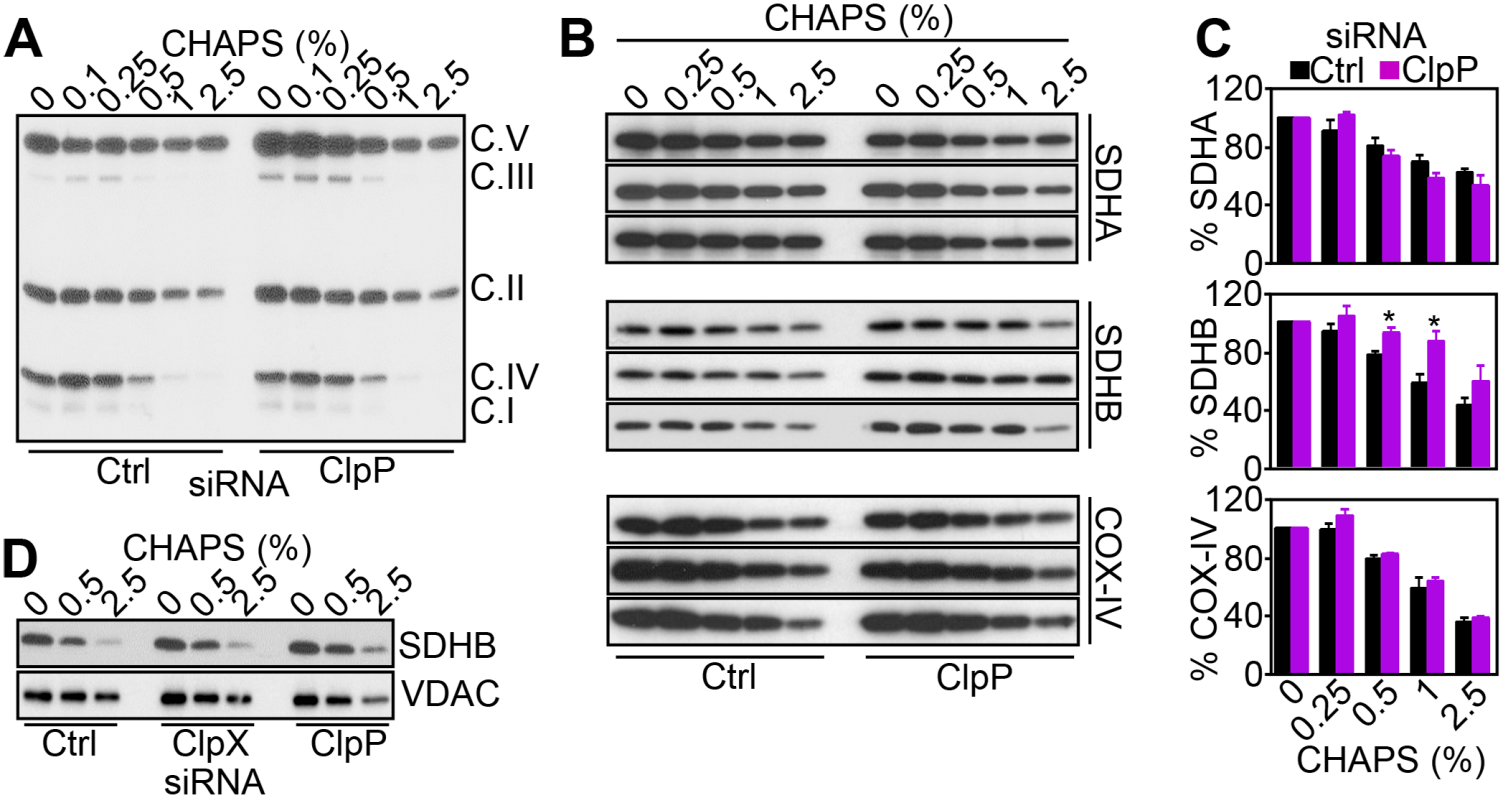
ClpXP regulates mitochondrial SDHB folding. (**A**) PC3 cells were transfected with control siRNA (Ctrl) or ClpP-directed siRNA and solubilized in the indicated concentrations of detergent (CHAPS), and insoluble fractions were analyzed by western blotting. The position of oxidative phosphorylation complex (C) subunits are indicated. (**B**) PC3 cells were transfected with control siRNA (Ctrl) or ClpP-directed siRNA and mixed with increasing concentrations of detergent (CHAPS), and insoluble fractions were analyzed by western blotting. Three independent experiments per condition are shown. (**C**) Densitometric quantification of protein bands in control or ClpP siRNA transfectants, as in (**B**). Data are the mean ± SEM of four (SDHB) or three (SDHA, COX-IV) independent experiments. (**D**) PC3 cells transfected with control siRNA (Ctrl) or ClpP-or ClpX-directed siRNA were mixed with the indicated increasing concentrations of CHAPS, and insoluble material was analyzed by western blotting. Raw data for this figure can be found in S1 Data.

In mitochondria, survivin associates with the oxidative phosphorylation Complex II subunit, SDHB, and regulates its stability [20]. Therefore, a role of ClpXP in this process [15] was next investigated. First, siRNA silencing of ClpP resulted in increased accumulation of SDHB in mitochondria, whereas COX-IV levels were not affected (S2A Fig). We next looked at the folding status of SDHB in these conditions. We found that siRNA silencing of ClpP caused the accumulation of detergent-insoluble, i.e., misfolded, mitochondrial Complex II, whereas subunits of oxidative phosphorylation Complex I, III, IV, or V were not affected (Fig 2A). When individual Complex II subunits were examined, ClpP knockdown selectively induced misfolding and aggregation of SDHB at different detergent concentrations (Figs 2B, 2C and S2B). In contrast, ClpP depletion did not affect the solubility of SDHA or other mitochondrial proteins, including COX-IV (Fig 2A and 2B), VDAC, or citrate synthase (CS; S2B Fig), compared to control transfectants. Aggregation and misfolding of SDHB, but not SDHA, after ClpP knockdown was observed under different detergent conditions (NP-40; S2C and S2D Fig). In addition, siRNA silencing of ClpX similarly induced accumulation of aggregated or misfolded SDHB (Figs 2D and S2E).

### ClpXP Is Required for Mitochondrial Respiration

**Fig 3:**
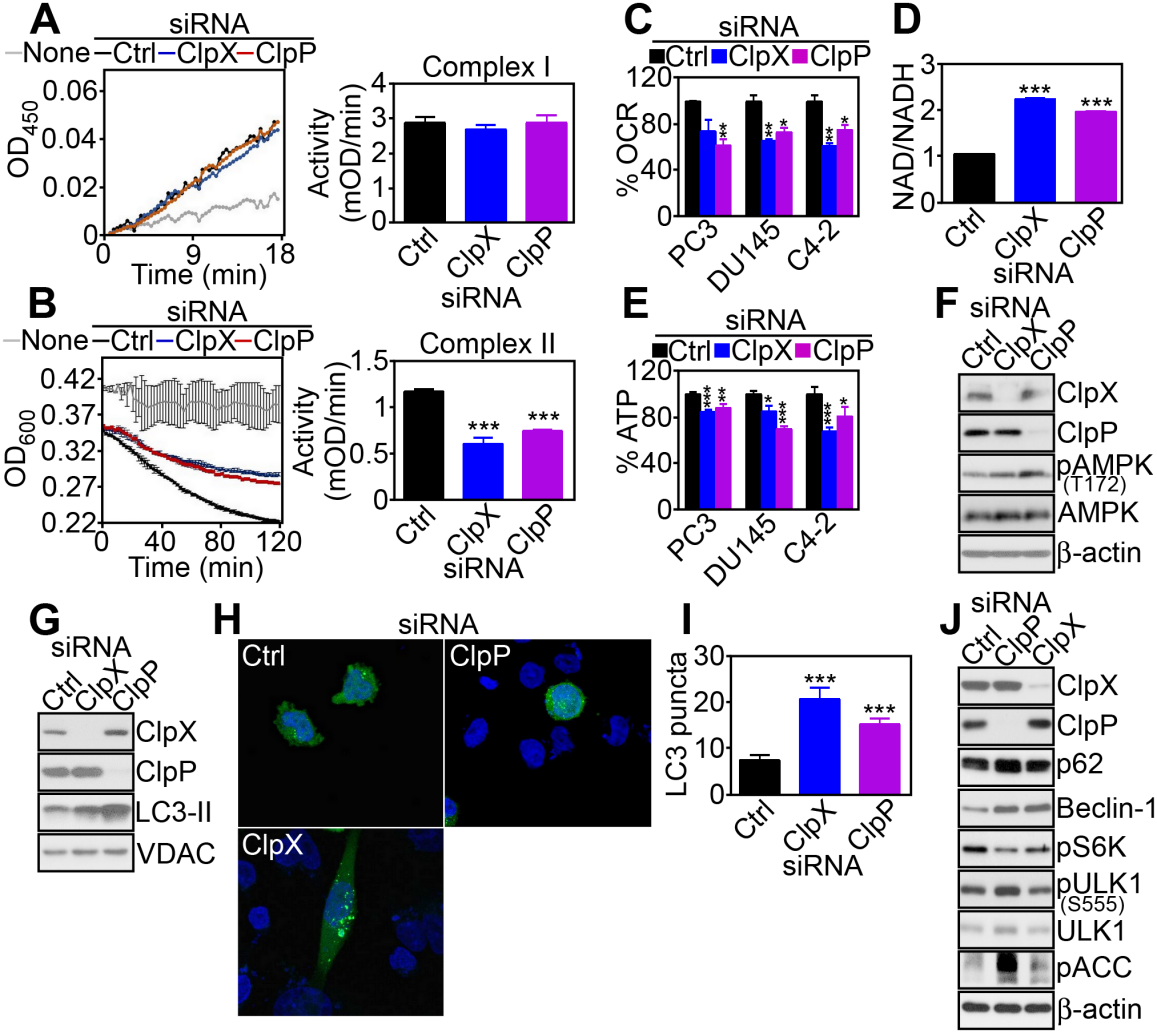
ClpXP regulation of mitochondrial bioenergetics. (**A** and **B**) PC3 cells were transfected with control siRNA (Ctrl), ClpX- or ClpP-directed siRNA, and mitochondrial extracts were analyzed for Complex I (**A**) or Complex II (**B**) activity. *Right*, Quantification of citrate synthase-normalized mitochondrial complex activity. ***, *p* < 0.0001. (**C–E**) The indicated prostate cancer cell types were transfected with control siRNA (Ctrl), ClpX- or ClpP-directed siRNA, and analyzed for changes in oxygen consumption rate (OCR, **C**), NAD/NADH ratio (PC3 cells, **D**), or ATP production (**E**). **, *p* < 0.006; ***, *p* < 0.0001. (**F** and **G**) PC3 cells transfected with the indicated siRNAs were analyzed by western blotting. (**H** and **I**) siRNA-silenced PC3 cells and transfected with GFP-LC3 were analyzed by fluorescence microscopy (**H**) and the number of LC3 puncta per cell were quantified (**I**). ***, *p* < 0.0001. (**J**) PC3 cells were transfected with control siRNA (Ctrl), CLpX- or ClpP-directed siRNA, and analyzed by western blotting. Raw data for this figure can be found in S2 Data.

Next, we asked whether SDHB misfolding induced by ClpXP targeting affected mitochondrial respiration. siRNA silencing of ClpX or ClpP did not affect mitochondrial Complex I activity in PC3 cells, compared to control siRNA transfectants (Fig 3A). In contrast, knockdown of ClpX or ClpP significantly reduced Complex II activity in PC3 cells (Fig 3B) as well as other prostate cancer cell types, including C4-2 (S3A Fig) and DU145 (S3B Fig). This response was specific because ClpX or ClpP targeting had no effect on mitochondrial Complex III (S3C Fig) or Complex IV (S3D Fig) activity, whereas Complex V function was increased in ClpP- but not ClpX-silenced PC3 cells (S3E Fig). Functionally, impaired Complex II activity after ClpXP targeting reduced oxygen consumption (Fig 3C), increased the NAD/NADH ratio (Figs 3D and S3F), and lowered overall ATP production (Fig 3E) in prostate cancer cells. Markers suggestive of compensatory glycolysis, including glucose consumption (S3G Fig) or lactate production (S3H Fig), were increased after ClpP- but not ClpX-silencing (S3G and S3H Fig). Consistent with defective bioenergetics, PC3 cells silenced for ClpP or ClpX exhibited increased phosphorylation of the energy sensor 5′ adenosine monophosphate-activated protein kinase (AMPK), whereas total AMPK levels were not affected (Fig 3F). In turn, AMPK phosphorylation coupled to downstream activation of autophagy, as determined by increased LC3-II conversion (Fig 3G), appearance of punctate LC3 staining by fluorescence microscopy (Fig 3H and 3I), accumulation of autophagy markers p62 and Beclin-1, and increased phosphorylation of ULK1 on its AMPK target site, Ser555 (Fig 3J).

### Targeting Mitochondrial Proteases Induces Oxidative Stress

**Fig 4:**
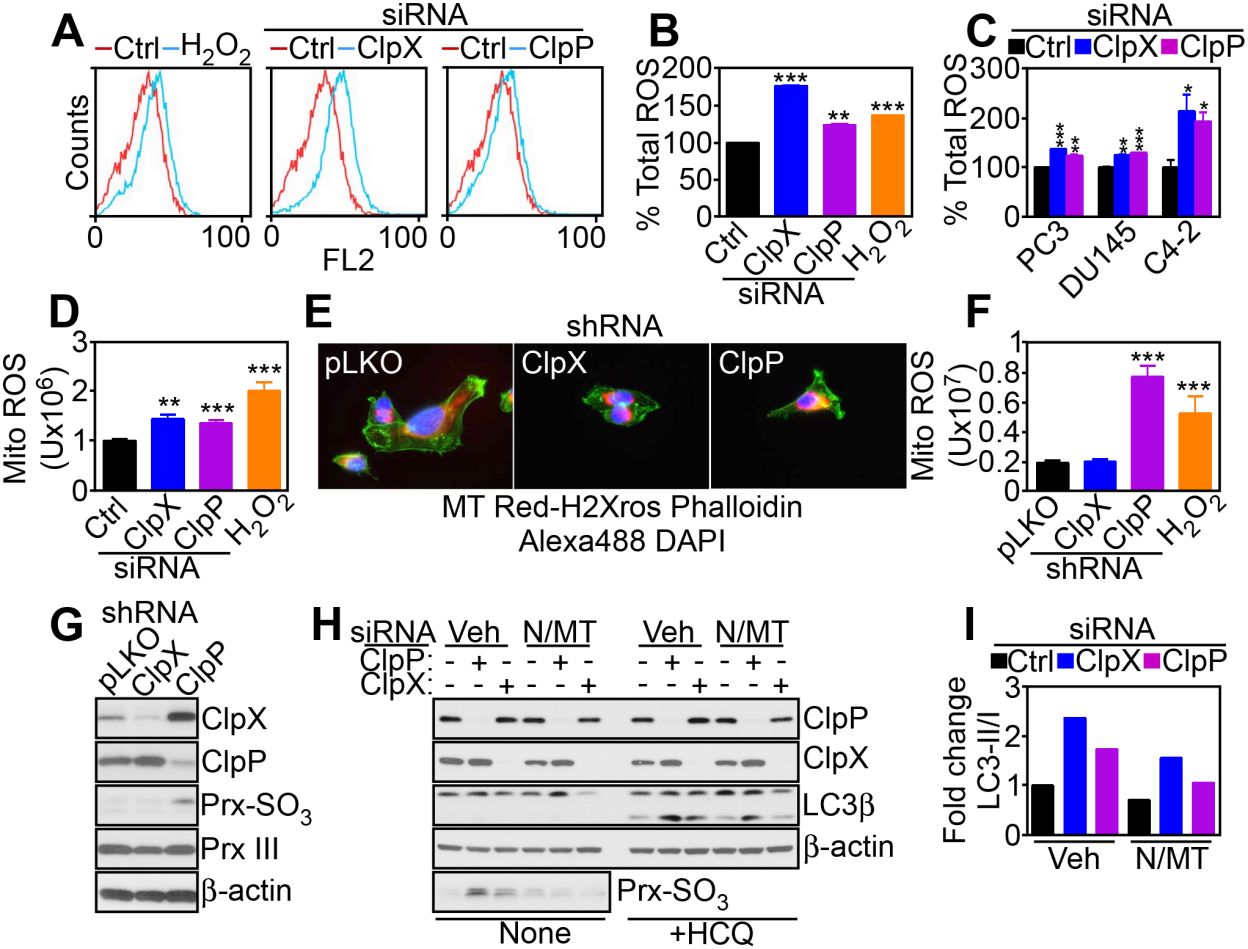
Mitochondrial ROS modulation by ClpXP. (**A** and **B**) PC3 cells transfected with control siRNA (Ctrl) or ClpX- or ClpP-directed siRNA were labeled with CellROX Green Reagent by flow cytometry (**A**), and staining intensity was quantified (**B**). H_2_O_2_ was a control oxidative stimulus. **, *p* = 0.004; ***, *p* < 0.0001. (**C**) The indicated prostate cancer cell types were analyzed for total ROS production as in (**A**). *, *p* = 0.03–0.04; **, *p* = 0.001; ***, *p* = 0.0008–<0.0001. (**D**) PC3 cells transfected with the indicated siRNAs as in (**A**) were analyzed for MitoSOX red mitochondrial superoxide reactivity by fluorescence microscopy and quantified. H_2_O_2_ was a control oxidative stimulus. **, *p* = 0.001; ***, *p* = 0.0004–0.0001. (**E** and **F**) PC3 cells stably transduced with control pLKO or shRNA to ClpX or ClpP were analyzed for mitochondrial superoxide production by fluorescence microscopy (**E**), and staining intensity was quantified (**F**). ***, *p* = 0.0004–0.0001. (**G**) PC3 cells stably transduced with pLKO or ClpX- or ClpP-directed shRNA were analyzed by western blotting. (**H**) PC3 cells transfected with control siRNA or ClpX- or ClpP-directed siRNA were incubated with vehicle (Veh) or the combination of antioxidants NAC (N) plus mitochondrial-directed MitoTempo (MT) and analyzed by western blotting in the absence or presence of the autophagic flux inhibitor hydroxychloroquine (HCQ). (**I**) Densitometric quantification of LC3-II/I ratio in vehicle (Veh) or antioxidant-treated PC3 cells in (**H**). Raw data for this figure can be found in S3 Data.

Based on these data, we next looked at the downstream consequences of defective mitochondrial respiration in ClpXP-targeted cells. siRNA silencing of ClpP or ClpX resulted in increased total cellular superoxide production in prostate cancer cells (Fig 4A–4C). This response was also associated with heightened production of mitochondria-specific reactive oxygen species (ROS), compared to control transfectants (Fig 4D). To rule out potential off-target effects, we next generated clones of PC3 and DU145 cells with stable shRNA knockdown of ClpP or ClpX (S4A Fig). Stable depletion of ClpXP did not affect total mitochondrial content (S4B Fig) or mitochondrial membrane potential (S4C Fig). In contrast, stable knockdown of ClpP increased mitochondrial ROS production (Fig 4E and 4F), with hyperoxidation of mitochondrial peroxiredoxin III (Prx III), a marker of oxidative damage (Fig 4G). Total Prx III levels were not affected (Fig 4G). In contrast, stable shRNA silencing of ClpX did not significantly modulate mitochondrial ROS production (Fig 4E and 4F) or Prx III hyperoxidation (Fig 4G), suggesting that other mechanisms compensated for ClpX loss in the stable cell line. Treatment with the antioxidant N-acetylcysteine (NAC) or mitochondrial ROS scavenger MitoTempo alone did not rescue Prx III hyperoxidation after ClpXP knockdown (S4D Fig). Conversely, the combination of NAC plus MitoTempo reversed the hyperoxidation of Prx III in these cells (S4D Fig). Functionally, we next asked if oxidative stress induced by ClpXP targeting was important for autophagy induction in these settings. Consistent with this possibility, the combination of NAC plus MitoTempo significantly attenuated LC3-II conversion in ClpP- or ClpX-silenced cells (Fig 4H and 4I), and this response was amplified by the autophagic flux inhibitor hydroxychloroquine (Fig 4H).

### ClpXP Overexpression in Human Cancer

**Fig 5:**
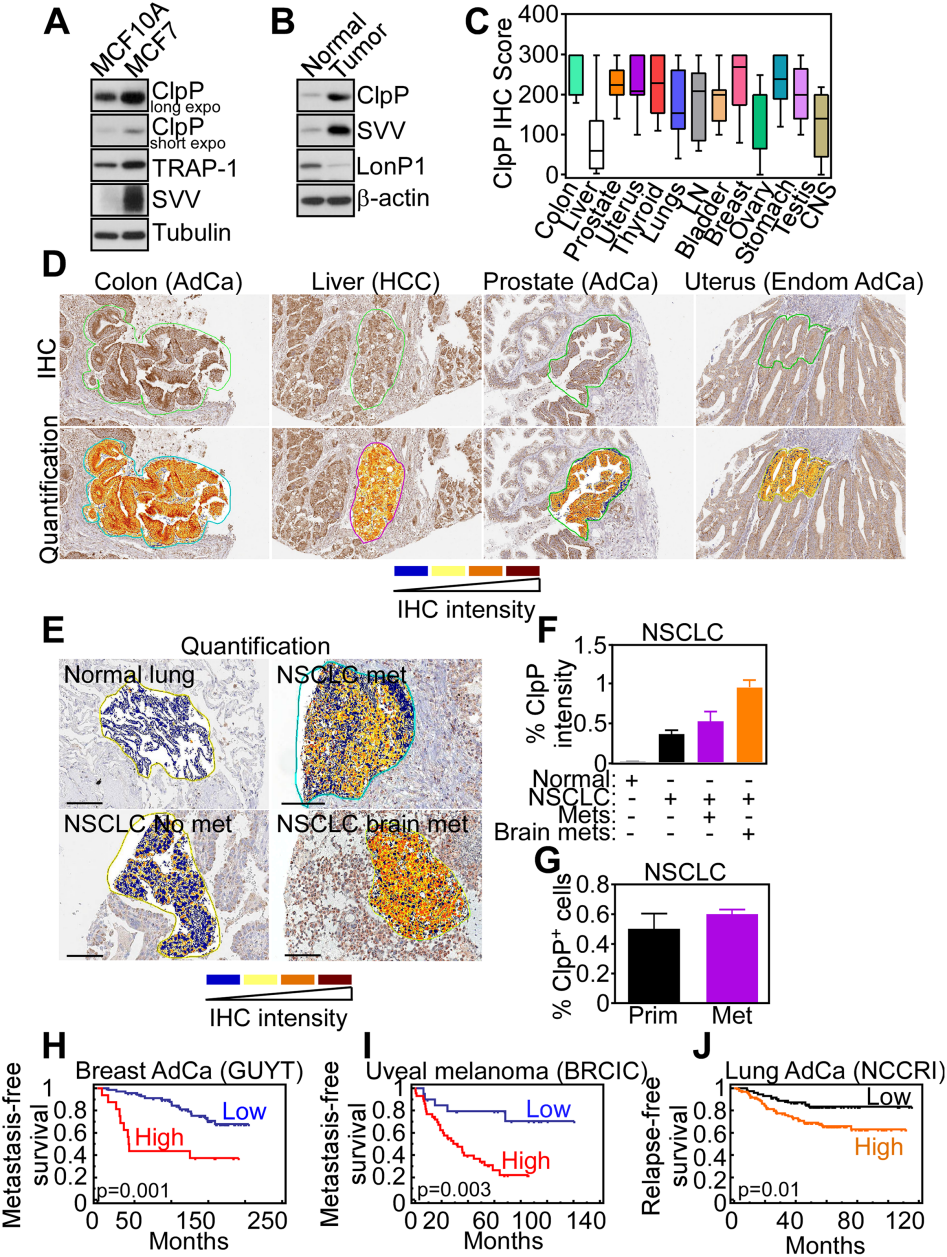
ClpP expression in human cancer. (**A** and **B**) Breast adenocarcinoma MCF-7 cells, non-tumorigenic breast epithelial MCF-10A cells (**A**), or primary human tissue samples representative of normal prostate or prostatic adenocarcinoma (**B**) were analyzed by western blotting. (**C**) Immunohistochemistry (IHC) score of ClpP expression in the indicated primary human tumors arrayed in a universal cancer tissue microarray. LN, lymph nodes; CNS, central nervous system. (**D**) IHC staining and Aperio quantification of ClpP expression in the indicated human tumor types. AdCa, adenocarcinoma; HCC, hepatocellular carcinoma; Endom, endometrial AdCa. (**E**) Aperio quantification of ClpP IHC expression in primary tissue samples of non-small cell lung cancer (NSCLC) that developed (met) or did not develop (no met) distant metastases or brain metastases (brain met) during a 5-y follow-up. (**F**) Quantification of ClpP staining intensity in the NSCLC cases in (**E**). (**G**) Quantification of ClpP-positive cells in primary and brain metastatic NSCLC. (**H–J**) Kaplan-Meier curves of metastasis-free survival (**H** and **I**) or relapse-free survival (**J**) in the indicated patient series with high or low expression of ClpP. AdCa, adenocarcinoma. Raw data for this figure can be found in S4 Data.

To complement the results obtained with tumor cell lines, we next looked at a potential differential expression of ClpXP in human cancer. First, ClpP was prominently upregulated in breast adenocarcinoma MCF-7 cells, compared to non-tumorigenic breast epithelial MCF-10A cells (Fig 5A). Similarly, ClpP was highly expressed in primary tissue samples of human prostatic adenocarcinoma, but not normal prostate epithelium (Fig 5B). For comparison, another protease involved in mitochondrial protein quality control, LonP1, was expressed in normal prostate but not prostate cancer (Fig 5B). When applied to tissue extracts of patient-derived tumor samples, an antibody to ClpP reacted with a predominant single band by western blotting (S5A Fig), reinforcing its specificity. Immunohistochemical staining of a universal cancer tissue microarray demonstrated that ClpP was overexpressed in virtually every human malignancy examined (Fig 5C), with intense cytoplasmic staining in the tumor cell population (Figs 5D and S5B). ClpP expression in cancer patients was independent of grade (colon adenocarcinoma and CNS tumors), Gleason score (prostate adenocarcinoma), histotype (lung cancer), or aggressive versus indolent lymphomas (S5C Fig). Conversely, ClpP levels were increased in histotypes of breast adenocarcinoma compared to normal epithelium (S5C Fig). In addition, ClpP became more prominently expressed in metastatic non-small cell lung cancer (NSCLC), compared to non-metastatic lesions, with the highest levels in brain-metastatic NSCLC (Figs 5E, 5F and S5D). In contrast, no difference in the percentage of ClpP-positive cells was observed in primary or metastatic NSCLC (Fig 5G). Consistent with these results, bioinformatics meta-analysis of public databases (PrognoScan) revealed that ClpP expression correlated with poorer outcome in 9 out of 14 analyzed datasets (64%; S2 Table and Fig 5H–5J), whereas ClpP levels were associated with better prognosis (S2 Table) in only one dataset (Melbourne). Importantly, high levels of ClpP expression were associated with shortened distant metastasis-free survival (S2 Table) in patients with breast adenocarcinoma (Fig 5H) and uveal melanoma (Fig 5I) and in abbreviated relapse-free survival in lung adenocarcinoma (Fig 5J).

### Regulation of Tumor Cell Proliferation by ClpXP

**Fig 6:**
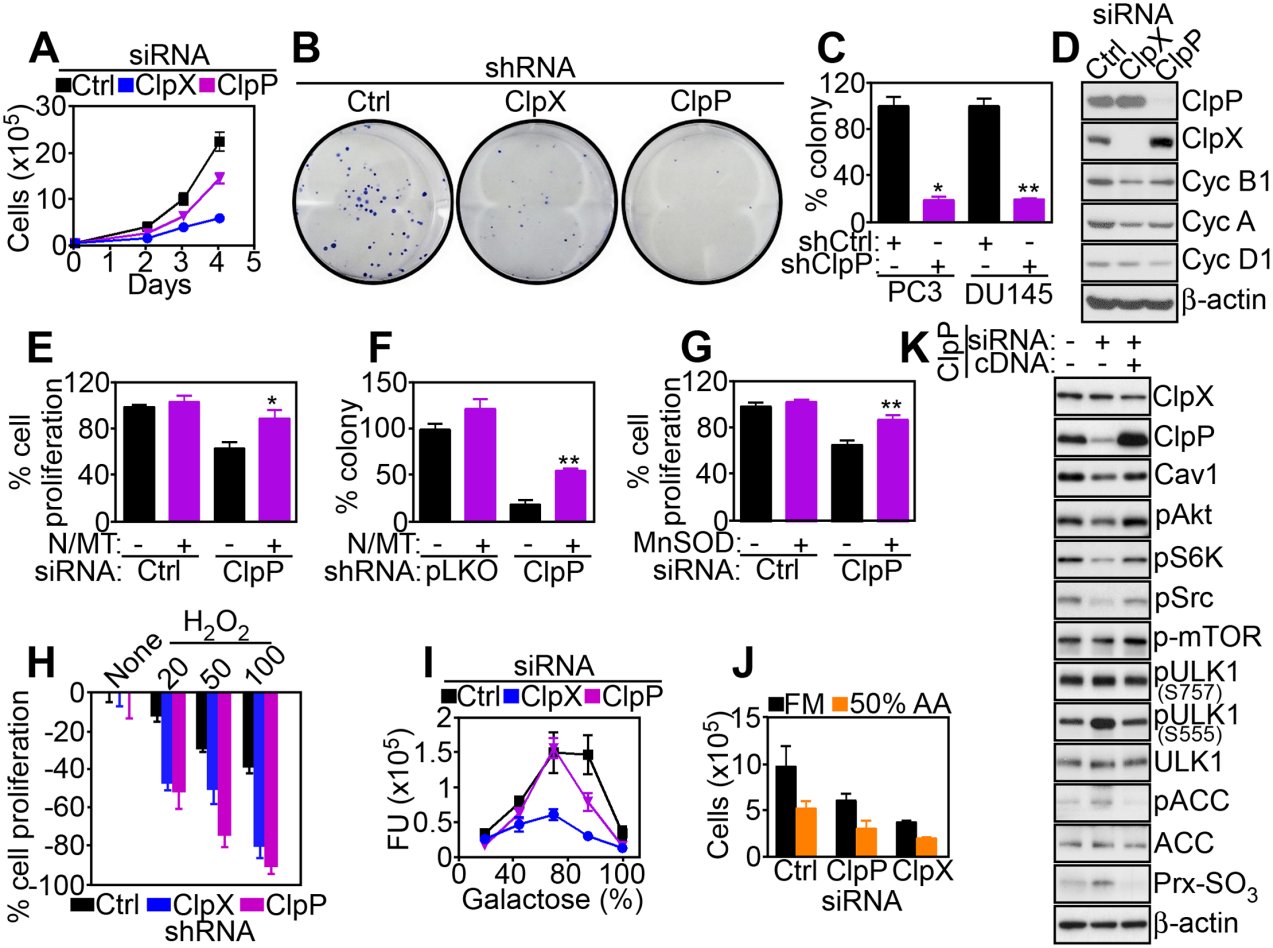
ClpXP regulation of tumor cell proliferation. (**A**) PC3 cells were transfected with control siRNA (Ctrl) or ClpX- or ClpP-directed siRNA and analyzed for cell proliferation at the indicated time intervals by direct cell counting. (**B** and **C**) PC3 or DU145 cells stably transduced with pLKO or ClpX- or ClpP-directed shRNA were analyzed for colony formation by crystal violet staining after 7 d (**B**, PC3) and quantified (**C**). *, *p* = 0.01; **, *p* = 0.006. (**D**) PC3 cells transfected with the indicated siRNAs as in (**A**) were analyzed by western blotting. (**E** and **F**) PC3 cells transfected with the indicated siRNAs (**E**) or shRNAs (**F**) were incubated with the combination of antioxidants NAC (N) plus mitochondrial-directed MitoTempo (MT) and analyzed for cell proliferation by direct cell counting (**E**) or colony formation (**F**). *, *p* = 0.028; **, *p* = 0.0026. (**G**) PC3 cells treated with control siRNA (Ctrl) or ClpP-directed siRNA were transfected with vector or MnSOD cDNA, and analyzed for cell proliferation by direct cell counting. **, *p* = 0.001. (**H**) PC3 cells stably transduced with pLKO or ClpX- or ClpP-directed shRNA were incubated with the indicated increasing concentrations of H_2_O_2_ (mM) and analyzed for inhibition of cell proliferation by direct cell counting. (**I** and **J**) PC3 cells transfected with the indicated siRNAs were incubated with increasing galactose:glucose ratios (**I**) or amino acid-deprived medium (50% amino acids, **J**) and analyzed for cell proliferation by direct cell counting. FU, fluorescence units. (**K**) PC3 cells transfected with ClpP-directed siRNA were reconstituted with ClpP cDNA and analyzed by western blotting. Raw data for this figure can be found in S5 Data.

Based on these results, we next asked how ClpXP influenced tumor progression. In a first series of experiments, knockdown of ClpP or ClpX partially reduced tumor cell proliferation (Figs 6A and S6A) and inhibited colony formation (Figs 6B, 6C and S6B), a marker of tumorigenicity. The effect of ClpXP silencing on tumor cell proliferation was cell-type-specific and more pronounced after knockdown of ClpP compared to ClpX (S6A Fig). In addition, silencing of ClpP or ClpX minimally reduced proliferation of non-metastatic breast adenocarcinoma MCF-7 cells, and non-tumorigenic breast epithelial MCF-10A cells were not affected (S6A Fig). When characterized in sensitive PC3 cells, knockdown of ClpXP resulted in lower levels of cyclins A, B1, and D1 (Fig 6D), reduced number of BrdU-positive cells (S6C Fig), and accumulation of cells with G1 DNA content (S6D Fig), consistent with cell cycle arrest.

Next, we looked at the mechanism(s) of ClpXP regulation of tumor cell proliferation. First, silencing of ClpP or ClpX in MCF-7 cells had little to no effect on mitochondrial Complex II activity (S6E Fig) and oxygen consumption rate (OCR, S6F Fig), thus mirroring the marginal sensitivity of these cells to ClpXP targeting (S6A Fig). In addition, ClpP or ClpXP knockdown did not reduce Complex II activity (S6G Fig) or OCR (S6H Fig) in non-tumorigenic MCF-10A cells, further linking impaired mitochondrial respiration induced by ClpXP targeting to reduced tumor cell proliferation. Based on these results, we next asked if aberrant ROS production associated with ClpXP targeting interfered with tumor cell proliferation. Consistent with this possibility, the combination of antioxidants NAC plus MitoTempo (MT) restored tumor cell proliferation (Fig 6E) and colony formation (Fig 6F) in ClpP-silenced PC3 cells. Similarly, transfection of the ROS scavenger MnSOD rescued the defect of cell proliferation induced by ClpP knockdown (Fig 6G). Finally, ClpXP depletion further sensitized tumor cells to “stress stimuli,” with more sustained inhibition of tumor cell proliferation mediated by oxidative stress (H_2_O_2_, Fig 6H), high galactose:glucose ratios (Fig 6I), or amino acid deprivation (50% amino acids, Fig 6J). We next reconstituted ClpP-silenced PC3 cells with siRNA-insensitive ClpP cDNA to test the specificity of these findings. Re-expression of ClpP under these conditions rescued the phosphorylation of Akt, Src, and p70S6K in ClpP-silenced cells (Fig 6K). In addition, re-expression of ClpP reversed the induction of autophagy (S555 phosphorylation of ULK1), AMPK signaling (ACC phosphorylation), and oxidative stress (Prx III hyperoxidation) induced by ClpP silencing (Fig 6K).

### ClpXP Regulation of Tumor Cell Invasion and Metastasis

**Fig 7:**
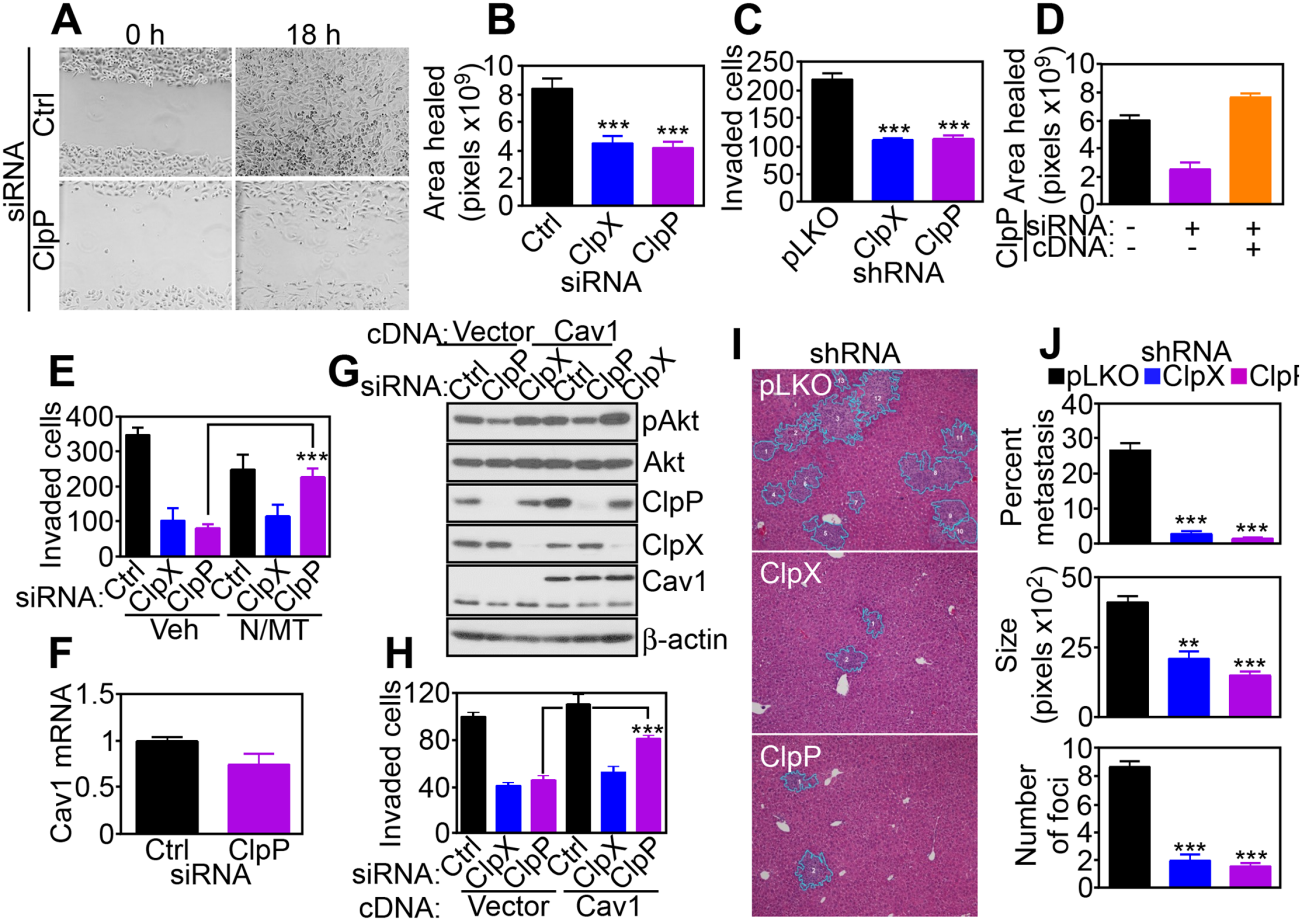
Requirement of ClpXP for tumor cell invasion and metastasis. (**A** and **B**) PC3 cells transfected with control siRNA (Ctrl) or ClpX- or ClpP-directed siRNA were analyzed for directional cell migration in a wound closure assay by light microscopy (ClpP, **A**), and wound surface area was quantified after 18 h (**B**). ***, *p* < 0.0001. (**C**) PC3 cells stably transduced with pLKO or ClpX- or ClpP-directed shRNA were analyzed for cell invasion across Matrigel-coated inserts. ***, *p* < 0.0001. (**D**) PC3 cells transfected with ClpP-directed siRNA were reconstituted with ClpP cDNA and analyzed for cell migration in a wound closure assay as in (**A** and **B**). (**E**) PC3 cells transfected with control siRNA (Ctrl) or ClpX- or ClpP-directed siRNA were analyzed for Matrigel invasion in the presence of vehicle (Veh) or the combination of antioxidants NAC (N) plus MitoTempo (MT). ***, *p* < 0.0001. (**F**) PC3 cells transfected with control siRNA (Ctrl) or ClpP-directed siRNA were analyzed for Caveolin-1 (Cav1) mRNA levels by quantitative PCR. (**G** and **H**) PC3 cells were transfected with the indicated siRNAs, reconstituted with vector or Cav1 cDNA, and analyzed by western blotting (**G**) or Matrigel invasion (**H**). ***, *p* < 0.0001. (**I**) PC3 cells stably transduced with pLKO or ClpX- or ClpP-directed shRNA were injected in the spleen of immunocompromised mice and livers collected after 11 d, which were analyzed by hematoxylin-eosin staining and light microscopy. (**J**) Quantification of metastatic burden (% metastasis, *top*), number of metastatic foci (*middle*), and size of metastatic foci (*bottom*) in mice intrasplenically injected with shRNA-transduced PC3 cells as in (**I**). **, *p* = 0.002; ***, *p* < 0.0001. Raw data for this figure can be found in S6 Data.

A potential participation of ClpXP in other tumor traits was investigated next, and we focused on cell motility, which requires mitochondrial bioenergetics [20]. siRNA knockdown of ClpP or ClpX inhibited directional PC3 cell migration in a wound closure assay (Fig 7A and 7B), and suppressed tumor cell invasion across Matrigel-coated Transwell inserts (Figs 7C and S7A). This response was specific because reconstitution of ClpP-silenced cells with a ClpP cDNA restored tumor cell motility in a wound closure assay (Fig 7D). Mechanistically, the combination of antioxidants NAC plus MitoTempo rescued the defect of tumor cell invasion mediated by ClpP knockdown (Fig 7E), demonstrating that increased ROS production in these settings was responsible for the inhibition of cell motility.

Next, we mapped the signaling requirements of ClpXP regulation of tumor cell motility. In these experiments, siRNA silencing of ClpP attenuated Akt (S473) phosphorylation (S7B Fig) and lowered the expression of cell motility effectors Caveolin-1 (Cav1) and Axl (S7B Fig). ClpXP knockdown was marginally effective (S7B Fig), and no changes in Cav1 mRNA levels were observed in control or ClpP siRNA transfectants (Fig 7F). Exposure of tumor cells to the oxidative stressor H_2_O_2_ mimicked this response, causing hyperoxidation of Prx III and loss of Cav1 expression (S7C Fig). Accordingly, siRNA silencing of Cav1 in PC3 cells was sufficient to reduce the levels of phosphorylated Akt (S473) (S7D Fig) and suppressed tumor cell migration and invasion, compared to control transfectants (S7E Fig). To validate a role of Cav1 in this cell motility pathway, we next reconstituted ClpP- or ClpX-depleted cells with a Cav1 cDNA. Re-expression of Cav1 in these settings restored Akt phosphorylation (Fig 7G) and rescued the defect of tumor cell invasion (Fig 7H) after ClpP, but not ClpX knockdown. In contrast, reconstitution of ClpXP-targeted cells with an Akt cDNA had no effect (S7F Fig).

Finally, we asked if ClpXP regulation of tumor cell motility was important for metastasis in vivo. Intra-splenic injection of PC3 cells transfected with control shRNA gave rise to extensive metastatic dissemination to the liver of immunocompromised mice within 11 d of injection (Fig 7I and 7J). In contrast, stable silencing of ClpX or ClpP in these cells suppressed the size, number, and extent of liver metastases at the same time interval (Fig 7I and 7J).

## Discussion

In this study, we have shown that the unfoldase-peptidase ClpXP forms a complex with survivin and the Hsp90-like chaperone TRAP-1 in mitochondria of tumor cells. This interaction maintains protein quality control and function of the oxidative phosphorylation Complex II subunit SDHB. Accordingly, genetic targeting of ClpXP causes the accumulation of misfolded SDHB, resulting in impaired bioenergetics, oxidative damage, and activation of stress signals, including autophagy. ClpXP is dramatically upregulated in primary and disseminated human tumors, correlates with shortened patient survival, and mechanistically supports tumor cell proliferation, cell motility, and heightened metastatic competence in vivo.

Extensively studied in bacteria [21] and proposed as a regulator of cell persistence [22], the ClpXP multimolecular complex [23] comprises a ClpX subunit that functions as an ATPase-directed unfoldase for unstructured protein substrates and an internal caseinolytic peptidase, ClpP, which degrades the translocated, unfolded peptides [14]. Although this proteasome-like arrangement is conserved in mammalian cells [15], the data presented here suggest that the ClpP and ClpX subunits may not have completely overlapping function(s) in tumor mitochondria, especially with respect to Akt activation and Cav1-dependent tumor cell motility. It is possible that these different responses reflect individual mitochondrial proteins independently regulated by ClpP or ClpX, or, alternatively, the coupling of individual ClpXP subunits to separate regulators of downstream signaling.

The interaction between TRAP-1 [10] and ClpXP [14] described here brings together the two main mechanisms of proteostasis: chaperone-regulated protein folding (TRAP-1) and proteolytic removal of misfolded molecules (ClpXP) in a single, functional continuum. A mitochondrial pool of survivin contributes to this proteostasis network, potentially as a scaffolding protein that binds both ClpP (this study) and TRAP-1 [20], and contributes to the stability of the complex. Consistent with the earlier elucidation of a TRAP-1 proteome in tumor mitochondria [10], a key substrate of the proteostasis network identified here was the iron-sulfur SDHB subunit of oxidative phosphorylation Complex II. The biochemical requirements of mitochondrial respiration and electron transport chain are well understood [24], but the possibility that these activities may depend on a carefully orchestrated balance of protein folding/protein clearance, especially in the proteotoxic-prone environment of tumor mitochondria [5], has not been widely considered. Consistent with this possibility, perturbation of the mitochondrial proteostasis network by TRAP-1 targeting [10] or ClpXP knockdown (this study) caused SDHB degradation or, conversely, accumulation of misfolded or aggregated SDHB. The exquisite specificity of this response, in which none of the other oxidative phosphorylation complexes are affected, highlights a potential unique propensity of SDHB to protein misfolding, especially in tumor mitochondria, or, alternatively, a more stringent requirement of protein quality control to enable efficient Complex II activity.

Irrespective, the loss of SDHB due to defective proteostasis profoundly affected mitochondrial bioenergetics, with decreased oxygen consumption, loss of ATP production, and a phenotype of cellular “stress,” characterized by activation of a mitochondrial and ER UPR [11], AMPK phosphorylation [25], stimulation of autophagy [12], and loss of mechanistic target of rapamycin (mTOR) signaling [20]. As shown here, an important mediator of these responses was the increased production of mitochondrial ROS and the ensuing oxidative damage likely associated with a defective electron transport chain [24]. This model is consistent with other data in the literature that homozygous deletion of TRAP-1 caused increased ROS production, DNA damage, and reduced cell proliferation [26], whereas TRAP-1 overexpression is protective against oxidative stress [27,28]. Here, antioxidants that include a mitochondrial ROS scavenger, MitoTempo, prevented the activation of autophagy and rescued the defects of cell proliferation and tumor cell invasion associated with ClpXP targeting, establishing a causal role of mitochondrial oxidative stress in these tumor traits.

Although a role of TRAP-1 in tumor progression is recognized [29], the possibility that ClpXP-directed proteostasis [14] may be also exploited in cancer has been proposed only recently [30]. In that study, the ClpXP subunit ClpP was found overexpressed in a subset of human acute myeloid leukemias, and pharmacologic or genetic targeting of ClpP impaired mitochondrial oxidative phosphorylation, resulting in leukemia cell killing [30]. The data presented here extend these observations and demonstrate that ClpXP-directed proteostasis is exploited in most human cancers, correlating with shortened patient survival. Where the two studies diverge, however, is in the mechanistic underpinning of the proposed pathway. Here, ClpXP was recognized as a pivotal component of a proteostasis network that, together with TRAP-1 [10] and survivin [20], ensures mitochondrial homeostasis in tumors. In our hands, and at variance with recent findings [30], targeting ClpXP only partially reduced tumor cell proliferation and in a cell-type-specific manner, with no measurable effect on tumor cell viability. Instead, we found that ClpXP was required to support directional tumor cell migration, invasion, and heightened metastatic dissemination in vivo. Mechanistically, this pathway involved increased phosphorylation of key cell motility kinases Akt and Src, and reconstitution experiments in ClpP-silenced cells identified the membrane microdomain adapter caveolin-1 [31] as a novel, oxidative, stress-regulated mediator of tumor cell motility.

As most epithelial tumors rewire their metabolism toward glycolysis [32], a role of mitochondrial bioenergetics in cancer has been controversial, and SDHB itself has been at times dubbed as a “tumor suppressor.” On the other hand, oxidative phosphorylation remains an important energy source in most cancers [33], fueling critical disease traits such as tumor repopulation after oncogene ablation [34] and drug resistance [35]. The data presented here reinforce this model and establish a key requirement of mitochondrial integrity for tumor cell motility and metastatic competence in vivo. This conclusion fits well with other evidence that oxidative phosphorylation is required for membrane lamellipodia dynamics, turnover of focal adhesion complexes, and phosphorylation of cell motility kinases [20,36], supporting tumor cell invasion and metastatic dissemination, in vivo [25,37]. In the context of a proteostasis network, ClpXP may contribute to this response by efficiently removing misfolded or aggregated SDHB molecules to preserve Complex II bioenergetics as well as buffering organelle oxidative stress.

In summary, we have shown that the unfoldase-peptidase complex ClpXP [14] is universally exploited in human cancer and contributes to a mitochondrial proteostasis network that controls metabolic reprogramming and downstream signals of tumor cell proliferation, motility, and metastatic competence in vivo. There is now considerable interest in targeting unique features of tumor metabolism, including mitochondrial functions [38], as a novel approach to cancer therapy. In this context, proof-of-concept studies have demonstrated that small molecule targeting of mitochondrial Hsp90s [25] or ClpXP [30] is feasible and produces potent anticancer activity in preclinical models. Together, this suggests that therapeutic inhibition of the mitochondrial proteostasis network described here may provide a viable strategy to disrupt key requirements of tumor progression.

## Materials and Methods

### Patient Samples

All patient-related studies were reviewed and approved by an institutional review board at Fondazione IRCCS Ca’ Granda Ospedale Maggiore Policlinico Milan, Italy. A cohort of 53 patients with single brain metastases who underwent surgical resection for curative purposes between 2010 and 2015 was retrieved from the archives of Fondazione IRCCS Ca’ Granda Ospedale Maggiore Policlinico (Milan, Italy) and arranged in a tissue microarray (TMA), as described [39]. The predominant primary cancer histotype of metastatic cases was non-small cell lung cancer (NSCLC, *n* = 44). Nineteen NSCLC patients for which complete 5-y follow-up records were available were included in the study. During follow-up, 9 NSCLC patients developed metastatic disease to the brain, whereas 10 patients had no evidence of metastasis (S1 Table). A Cancer Universal TMA (CaU-TMA) representative of 13 different cancer types (10 cases for each tumor type) was described previously [40].

### Mitochondrial Protein Folding

Mitochondrial protein folding assays were performed as previously described [10]. Briefly, mitochondrial fractions were isolated from PC3 cells transfected with control non-targeting siRNA or ClpP- or ClpX-directed siRNA after 72 h and suspended in equal volume of mitochondrial fractionation buffer containing increasing concentrations of NP-40 (0%, 0.05%, 0.1%, 0.2%, 0.5%, or 2%) or CHAPS (0%, 0.1%, 0.25%, 0.5%, 1%, or 2.5%). Samples were incubated for 25 min on ice with vortexing every 5 min, and detergent-insoluble protein aggregates were isolated by centrifugation at 20,000 g for 20 min, separated on SDS polyacrylamide gels, and analyzed by western blotting.

### Analysis of Bioenergetics

The various prostate cancer cell types were transfected with control non-targeting siRNA or ClpP- or ClpX-directed siRNA for 48 h or 72 h and analyzed for ATP generation (BioChain cat No. Z5030041) or oxygen consumption (ENZO Lifesciences cat. No. ENZ-51045-1), as described previously [10]. In other experiments, fresh culture medium containing dialyzed FBS was harvested after 2 h and examined for lactate production (Abcam cat No. ab65331). The quantification of NAD^+^ or NADH was measured by enzymatic NADH recycling assay according to the manufacturer’s instruction (BioVision Cat No. K337-100). Briefly, PC3 cells (6 x 10^5^) transfected with control non-targeting siRNA or ClpP- or ClpX-directed siRNA were harvested after 72 h, and cell pellets were disrupted by two cycles of freezing and thawing in NADH/NAD^+^ extraction buffer. Soluble fractions were collected by centrifugation at 20,000 g for 5 min and processed for removal of NADH-consuming enzymes using YM-10 (Millipore). For assessment of NADH content, the cycling assay was performed after decomposition of NAD^+^ by heating at 60°C for 25 min, whereas the decomposition step was omitted for determination of total NAD^+^/H content.

### Mitochondrial Oxidative Phosphorylation Complex Activity

Various prostate cancer cell types were analyzed for changes in oxidative phosphorylation complex activity using Abcam reagents (Cat. no. ab109721—Complex I, ab109908—Complex II, ab109905—Complex II/III, ab109909—Complex IV) and Cayman reagent (701000—Complex V) using isolated lysed mitochondria, as described [10]. Briefly, tumor cells were transfected with control non-targeting siRNA or ClpP-or ClpX-directed siRNA and validated for protein knockdown by western blotting, and 2 μg of mitochondrial extracts from each condition were assayed for citrate synthase (CS) activity (ScienCell). Aliquots of mitochondrial lysates with comparable CS activity were applied for determination of the individual mitochondrial complex function. Relative complex activities were calculated by determining the change in absorbance over time in the linear range of the individual measurements.

### ROS Analysis

To detect total ROS, ClpP- or ClpX-silenced prostate cancer cell types were incubated with 2.5 μM of CellROX Green Reagent (Invitrogen) for 30 min at 37°C, according to the manufacturer’s instructions. After three washes in PBS, pH 7.4, cells were harvested and analyzed on a FACS Calibur flow cytometer, with the CellROX Green Reagent signal in FL1. Intact cells were gated in the FSC/SSC plot to exclude small debris. The resulting FL1 data were plotted on a histogram. Superoxide production by mitochondria was visualized by fluorescence microscopy, as described previously [36]. Briefly, 1.5 x 10^4^ cells were grown on high optical quality 8-well μ-slides (Ibidi) and stained with MitoSOX Red mitochondrial superoxide indicator (5 μM, 10 min) in complete medium, followed by extensive washing in warm complete medium. Stained cells were imaged with a 40X objective on a Nikon TE300 inverted time-lapse microscope equipped with a video system containing an Evolution QEi camera and a time-lapse video cassette recorder. The atmosphere was equilibrated to 37°C and 5% CO_2_ in an incubation chamber. Phase and red fluorescence (TRITC filter cube, excitation wavelength: 532–554 nm, emission wavelength: 570–613 nm) images were captured. To quantitate superoxide levels, files were imported into Image J and masks were manually created around the periphery of the cell based on the phase image and subsequently applied to the TRITC channel to measure intensity. A minimum of 100 cells were analyzed in each independent experiment to obtain mean values.

### Cell Migration and Invasion

The various tumor cell types suspended in 0.1% BSA/RPMI and seeded (1 x 10^5^ cells) in the upper compartment of 8 μM pore diameter BD Transwell membranes (BD) were quantified for cell migration, as described [20]. For cell invasion, the Transwell membranes were coated with Matrigel. In all experiments, NIH3T3 conditioned medium was placed in the lower compartment as a chemoattractant [25]. After 18 h incubation at 37°C, the Transwell membranes from each insert were recovered and cells on the upper side (non-migratory) were scraped off the surface. Cells on the lower side of the membrane were fixed in methanol, rinsed in water, and mounted on glass slides with Vectashield medium containing DAPI (Vector Laboratories). Migrated cells on each membrane were counted in five different fields at 20x magnification by fluorescence microscopy.

### Liver Metastasis Model

All experiments involving animals were carried out in accordance with the Guide for the Care and Use of Laboratory Animals of the National Institutes of Health. Protocols were approved by the Institutional Animal Care and Use Committee (IACUC) at The Wistar Institute. A liver metastasis models was performed essentially as described previously [25]. Briefly, PC3 cells stably transfected with control pLKO or ClpP- or ClpX-directed shRNA at 80% confluency were suspended in PBS, pH 7.4, and 50 μl containing 1 × 10^6^ cells were injected in the spleen of 6–8 wk-old male NOD SCID gamma (NSG, NOD.Cg-Prkdcscid Il2rgtm1Wjl/SzJ) mice (Jackson Laboratory). Spleens were removed the first day after injection to minimize potentially confounding effects on metastasis due to variable growth of primary tumors. Animals were sacrificed 11 d after injection of the tumor cells, and their livers were resected, fixed in formalin, and paraffin-embedded. Serial liver sections 500 μm apart (*n* = 15 per each condition) were stained with hematoxylin and eosin and analyzed histologically. Metastatic foci were quantified by morphometry and expressed as number and surface areas of metastatic tumor growth compared to total surface area, as described [25].

### Statistical Analysis

Data were analyzed using the two-sided unpaired *t* or chi-square tests using a GraphPad software package (Prism 6.0) for Windows. Data are expressed as mean ± SD or mean ± SEM of replicates from a representative experiment out of at least two or three independent determinations. A *p* value of <0.05 was considered statistically significant.

## Supporting Information

S1 Data Excel file containing the raw data for Fig 2. (XLSX)

S2 Data Excel file containing the raw data for Fig 3. (XLSX)

S3 Data Excel file containing the raw data for Fig 4. (XLSX)

S4 Data Excel file containing the raw data for Fig 5. (XLSX)

S5 Data Excel file containing the raw data for Fig 6. (XLSX)

S6 Data Excel file containing the raw data for Fig 7. (XLSX)

S7 Data Excel file containing the raw data for S1 Fig. (XLSX)

S8 Data Excel file containing the raw data for S2 Fig. (XLSX)

S9 Data Excel file containing the raw data for S3 Fig. (XLSX)

S10 Data Excel file containing the raw data for S4 Fig. (XLSX)

S11 Data Excel file containing the raw data for S5 Fig. (XLSX)

S12 Data Excel file containing the raw data for S6 Fig. (XLSX)

S13 Data Excel file containing the raw data for S7 Fig. (XLSX)

S1 Fig Mitochondrial survivin regulation of ClpP. (**A** and **B**) PC3 cells were treated with vehicle (Veh) or small molecule survivin (SVV) suppressant YM155, incubated with cycloheximide (CHX), and ClpP or SVV protein bands detected by western blotting after CHX release (**A**) was quantified by densitometry (**B**). (**C** and **D**) The experimental conditions are as in (**A** and **B**) except that PC3 cells were transfected with control siRNA (Ctrl) or SVV-directed siRNA and protein bands detected by western blotting after CHX release (**C**) was quantified by densitometry (**D**). (**E**) PC3 cells were transfected with control non-targeting siRNA (Ctrl) or SVV-directed siRNA and analyzed for ClpP mRNA levels by quantitative PCR. Raw data for this figure can be found in S7 Data.
(TIF)

S2 Fig Analysis of protein folding. (**A**) PC3 cells were transfected with control siRNA (Ctrl) or ClpP-directed siRNA and analyzed by western blotting. (**B**) PC3 cells transfected with control siRNA (Ctrl) or ClpP-directed siRNAs, as in (**A**), were solubilized in the indicated increasing concentrations of detergent (CHAPS), and insoluble (*top*) or soluble (*bottom*) fractions were analyzed by western blotting. CS, citrate synthase. (**C** and **D**) PC3 cells transfected with control siRNA (Ctrl) or ClpP-directed siRNA were solubilized in the indicated increasing concentrations of detergent (NP-40) and detergent-insoluble bands were visualized by western blotting (**C**) with quantification by densitometry (**D**). (**E**) PC3 cells were transfected with control non-targeting siRNA (Ctrl) or ClpP- or ClpX-directed siRNA and detergent-insoluble SDHB bands visualized by western blotting were quantified by densitometry at the indicated detergent (CHAPS) concentrations. Raw data for this figure can be found in S8 Data.
(TIF)

S3 Fig ClpXP regulation of mitochondrial respiration. (**A** and **B**) Prostate cancer C4-2 (**A**) or DU145 (**B**) cells were transfected with control non-targeting siRNA (Ctrl) or ClpP- or ClpX-directed siRNA and analyzed for Complex II activity. *Right*, quantification of citrate synthase-normalized Complex II activity. *, *p* = 0.01; **, *p* = 0.008. (**C–E**) PC3 cells transfected with control siRNA (Ctrl) or ClpP- or ClpX-directed siRNA were analyzed for mitochondrial Complex III (**C**), Complex IV (**D**), or Complex V (**E**) activity. *Right*, Quantification of citrate synthase-normalized mitochondrial complex activities. *, *p* = 0.04. (**F**) siRNA-transfected PC3 cells, as in (**C–E**), were analyzed for NAD/NADH ratio. (**G** and **H**) PC3 cells transfected with the indicated siRNAs were analyzed for glucose consumption (**G**) or lactate production (**H**). ***, *p* < 0.0001. Raw data for this figure can be found in S9 Data.
(TIF)

S4 Fig Characterization of stable cell lines. (**A**) PC3 cells were infected with control pLKO or shRNA directed to ClpX or ClpP and selected in puromycin-containing medium, and the indicated clones were analyzed by western blotting. (**B** and **C**) Control pLKO-transfectants or ClpX (clone #59) or ClpP (clone #59) shRNA transfectants were analyzed for total mitochondrial content (**B**) or changes in mitochondrial membrane potential (**C**) by TMRM labeling and flow cytometry. (**D**) PC3 cells were transfected with control siRNA (Ctrl) or ClpX- or ClpP-directed siRNA, mixed with the ROS scavengers NAC (N) or MitoTempo (MT), alone or in combination, and analyzed by western blotting. Raw data for this figure can be found in S10 Data.
(TIF)

S5 Fig ClpP expression in human tumors. (**A**) Tissue extracts from brain metastasis of non-small cell lung cancer (NSCLC) were separated by SDS gel electrophoresis and analyzed with an antibody to ClpP by western blotting. Undiff, undifferentiated; SCC, squamous cell carcinoma; AdCa, adenocarcinoma. (**B**) Primary tissue samples representative of the indicated tumor diagnoses were stained with an antibody to ClpP and analyzed by immunohistochemistry (IHC). Quantification of cytosolic ClpP staining in the marked tissue areas was carried out using the Aperio software (Quantification). Ca, carcinoma, AdCa, adenocarcinoma; HL, Hodgkin’s Lymphoma; HG, high-grade; IDC, infiltrating ductal carcinoma; ILC, infiltrating lobular carcinoma; GBM, glioblastoma. (**C**) Correlation between ClpP immunohistochemical (IHC) staining in primary human tumors and tumor grade (Colon AdCa, CNS tumors), Gleason score (prostate AdCa), lymphoma subtype (DLBCL, diffuse large B cell lymphoma; Follicular, follicular lymphoma; Mantle, mantle cell lymphoma; HL, Hodgkin’s lymphoma); histotype (lung cancer or breast AdCa; CIS, carcinoma in situ; IDC, infiltrating ductal carcinoma; ILB, infiltrating lobular carcinoma). Mening, meningioma. Data are expressed as mean ± SEM of a ClpP IHC score per each tumor type examined. (**D**) Primary tissue samples representative of normal lung, non-small cell lung cancer (NSCLC) that developed (met) or not (no met) distant metastases during a 5-years follow-up, or metastatic NSCLC to the brain were analyzed for ClpP expression by immunohistochemistry (IHC), with quantification of marked areas by Aperio. Raw data for this figure can be found in S11 Data.
(TIF)

S6 Fig ClpXP regulation of tumor cell proliferation. (**A**) The indicated tumor cell types were transfected with control siRNA (Ctrl) or ClpX- or ClpP-directed siRNA and analyzed for changes in cell proliferation by direct cell counting. *, *p* = 0.013–0.015; **, *p* = 0.001–0.007; ***, *p* < 0.0001. (**B**) PC3 cells stably transfected with control pLKO or shRNA to ClpX or ClpP were analyzed for colony formation after 7 d by crystal violet staining. **, *p* = 0.001–0.004. (**C**) PC3 cells transfected with the indicated siRNAs were analyzed for BrdU incorporation by flow cytometry, and the percentage of BrdU^+^ cells was quantified. **, *p* = 0.004; ***, *p* < 0.0001. (**D**) PC3 cells transfected as in (**A**) were analyzed for DNA content by propidium iodide staining and flow cytometry, and the percentage of cells in each cell cycle phase was quantified. (**E**) Breast adenocarcinoma MCF-7 cells were transfected with control siRNA (Ctrl) or ClpP- or ClpX-directed siRNA and analyzed for Complex II activity. *Right*, Quantification of citrate synthase-normalized Complex II activity. **, *p* = 0.002. (**F**) MCF-7 cells transfected with the indicated siRNAs were analyzed for oxygen consumption rates (OCR). (**G**) Breast epithelial MCF-10A cells were transfected with control siRNA (Ctrl) or ClpP- or ClpX-directed siRNA and analyzed for Complex II activity. *Right*, Quantification of citrate synthase-normalized Complex II activity. (**H**) siRNA-transfected MCF-10A cells as in (**G**) were analyzed for oxygen consumption rates (OCR). Raw data for this figure can be found in S12 Data.
(TIF)

S7 Fig ClpXP requirement for tumor cell invasion. (**A**) DU145 or LN229 cells were transfected with control siRNAs or ClpX- or ClpP-directed siRNA and analyzed for Matrigel invasion. ***, *p* < 0.0001. (**B**) LN229 cells were transfected with the indicated siRNAs as in (**A**) and analyzed by western blotting. (**C**) PC3 cells were treated with the indicated increasing concentrations of H_2_O_2_ and analyzed by western blotting. (**D** and **E**) PC3 cells were transfected with control siRNA (Ctrl) or Caveolin-1 (Cav1)-directed siRNA and analyzed by western blotting (**D**) or changes in cell migration (Migr) or cell invasion (Inv) across Transwell membranes (**E**). ***, *p* < 0.0001. (**F**) PC3 cells transfected with control siRNA (Ctrl) or ClpX- or ClpP-directed siRNA were reconstituted with vector or Akt cDNA and analyzed for cell invasion in a Transwell assay. Raw data for this figure can be found in S13 Data.
(TIF)

S1 Methods Supporting materials and methods. (DOCX)

S1 Table Patients’ characteristics. NSCLC, non-small cell lung cancer; PNET, pulmonary neuroendocrine tumor; other cancers: breast adenocarcinoma (1), melanoma (1), colorectal adenocarcinoma (1), ovarian carcinoma (1).
(DOCX)

S2 Table Meta-analysis of ClpP prognostic implications. (DOCX)

## Abbreviations

AMPK: 5′ adenosine monophosphate-activated protein kinase
CS: citrate synthase
ER: endoplasmic reticulum
Hsp90: heat shock protein-90
IAP: inhibitor-of-apoptosis protein
mTOR: mechanistic target of rapamycin
NAC: N-acetylcysteine
NSCLC: non-small cell lung cancer
OCR: oxygen consumption rate
ROS: reactive oxygen species
SDHB: succinate dehydrogenase B
siRNA: small interfering RNA
TRAP-1: TNFR-associated molecule-1
UPR: unfolded protein response
VDAC: voltage-dependent anion channel
