# Supplementary figures and images for: The Mitochondrial Unfoldase-Peptidase Complex ClpXP Controls Bioenergetics Stress and Metastasis
Source: PLoS Biol. 2016 Jul 7;14(7):e1002507. doi: 10.1371/journal.pbio.1002507 (PMC4936714; doi:10.1371/journal.pbio.1002507)

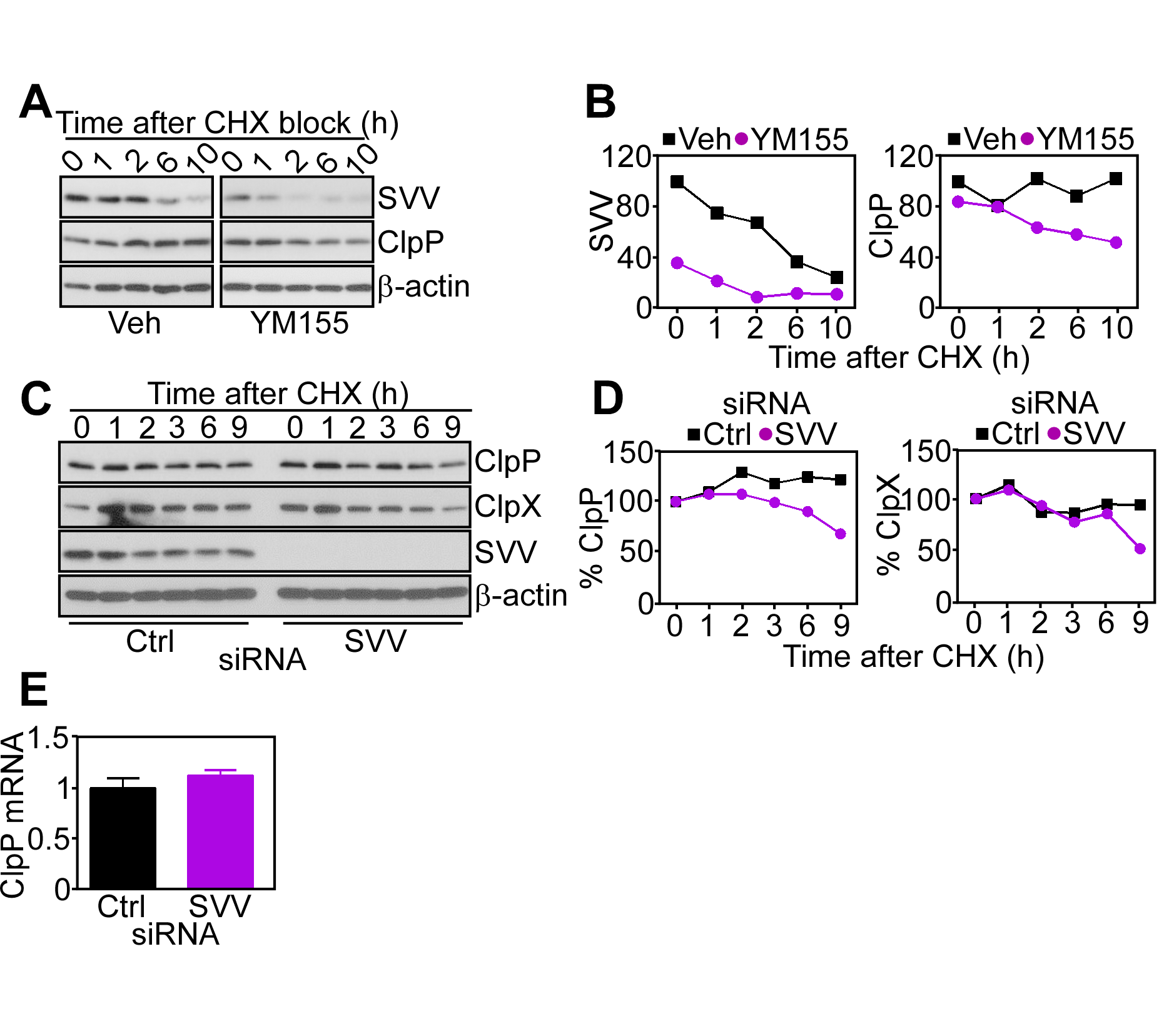

Supplement: S1 Fig — (A and B) PC3 cells were treated with vehicle (Veh) or small molecule survivin (SVV) suppressant YM155, incubated with cycloheximide (CHX), and ClpP or SVV protein bands detected by western blotting after CHX release (A) was quantified by densitometry (B). (C and D) The experimental conditions are as in (A and B) except that PC3 cells were transfected with control siRNA (Ctrl) or SVV-directed siRNA and protein bands detected by western blotting after CHX release (C) was quantified by densitometry (D). (E) PC3 cells were transfected with control non-targeting siRNA (Ctrl) or SVV-directed siRNA and analyzed for ClpP mRNA levels by quantitative PCR. Raw data for this figure can be found in S7 Data. (TIF) [file pbio.1002507.s014.tif]

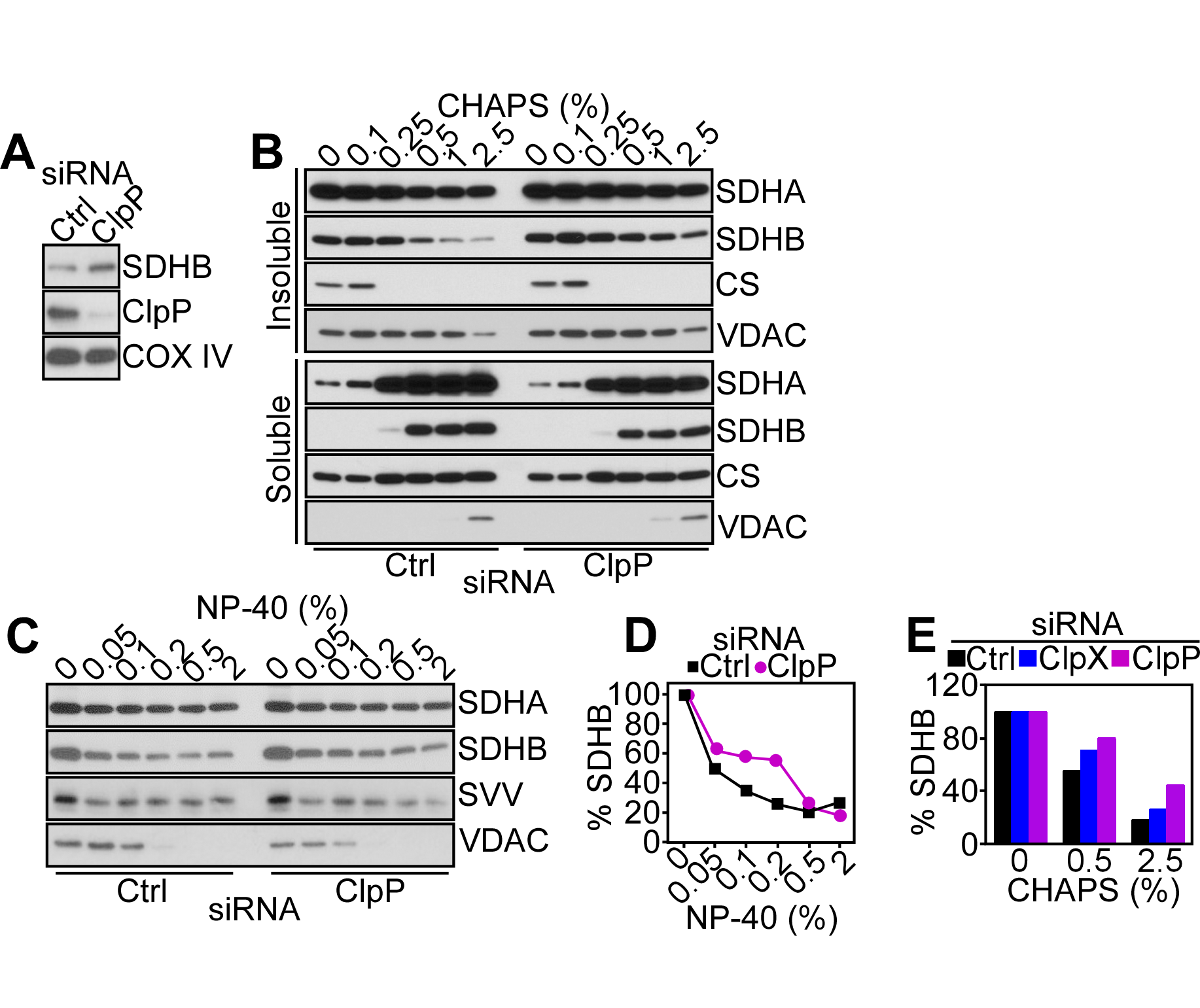

Supplement: S2 Fig — (A) PC3 cells were transfected with control siRNA (Ctrl) or ClpP-directed siRNA and analyzed by western blotting. (B) PC3 cells transfected with control siRNA (Ctrl) or ClpP-directed siRNAs, as in (A), were solubilized in the indicated increasing concentrations of detergent (CHAPS), and insoluble (top) or soluble (bottom) fractions were analyzed by western blotting. CS, citrate synthase. (C and D) PC3 cells transfected with control siRNA (Ctrl) or ClpP-directed siRNA were solubilized in the indicated increasing concentrations of detergent (NP-40) and detergent-insoluble bands were visualized by western blotting (C) with quantification by densitometry (D). (E) PC3 cells were transfected with control non-targeting siRNA (Ctrl) or ClpP- or ClpX-directed siRNA and detergent-insoluble SDHB bands visualized by western blotting were quantified by densitometry at the indicated detergent (CHAPS) concentrations. Raw data for this figure can be found in S8 Data. (TIF) [file pbio.1002507.s015.tif]

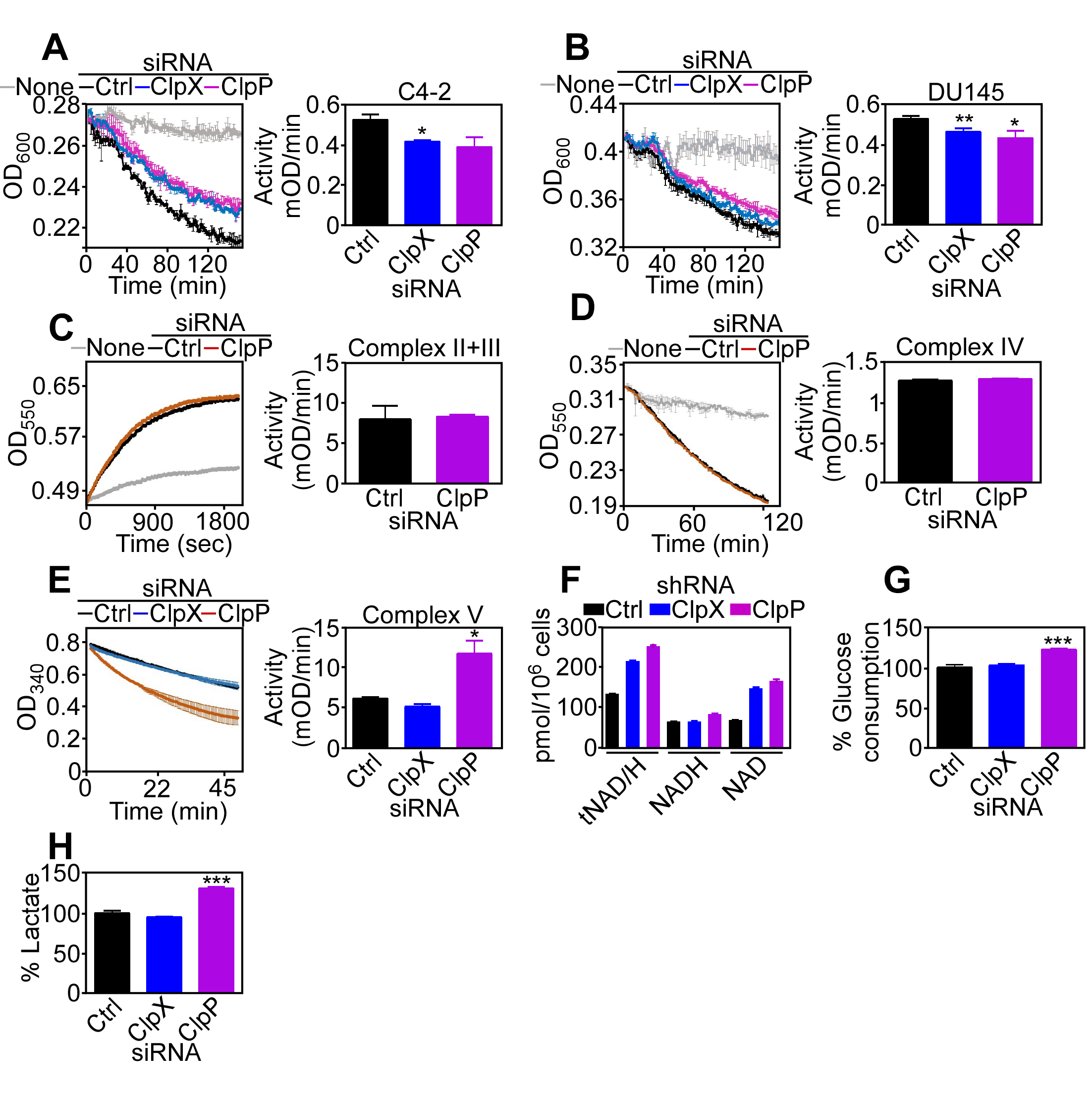

Supplement: S3 Fig — (A and B) Prostate cancer C4-2 (A) or DU145 (B) cells were transfected with control non-targeting siRNA (Ctrl) or ClpP- or ClpX-directed siRNA and analyzed for Complex II activity. Right, quantification of citrate synthase-normalized Complex II activity. *, p = 0.01; **, p = 0.008. (C–E) PC3 cells transfected with control siRNA (Ctrl) or ClpP- or ClpX-directed siRNA were analyzed for mitochondrial Complex III (C), Complex IV (D), or Complex V (E) activity. Right, Quantification of citrate synthase-normalized mitochondrial complex activities. *, p = 0.04. (F) siRNA-transfected PC3 cells, as in (C–E), were analyzed for NAD/NADH ratio. (G and H) PC3 cells transfected with the indicated siRNAs were analyzed for glucose consumption (G) or lactate production (H). ***, p < 0.0001. Raw data for this figure can be found in S9 Data. (TIF) [file pbio.1002507.s016.tif]

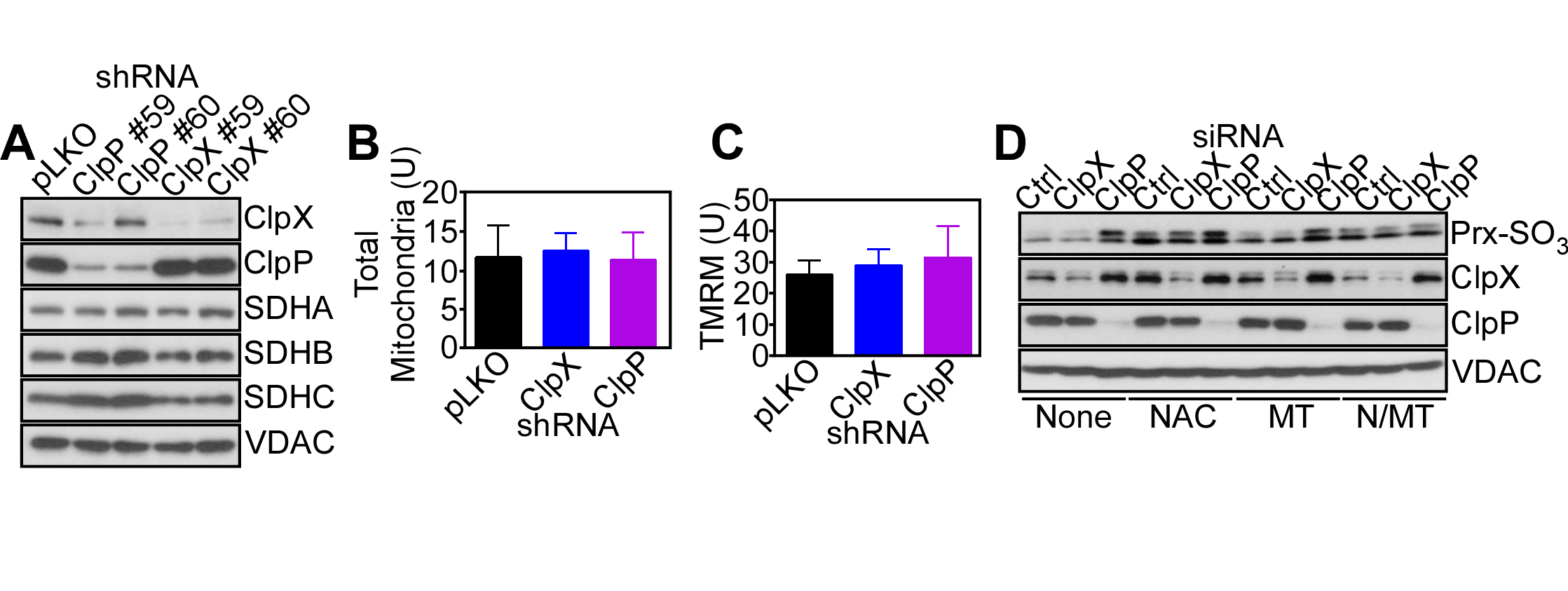

Supplement: S4 Fig — (A) PC3 cells were infected with control pLKO or shRNA directed to ClpX or ClpP and selected in puromycin-containing medium, and the indicated clones were analyzed by western blotting. (B and C) Control pLKO-transfectants or ClpX (clone #59) or ClpP (clone #59) shRNA transfectants were analyzed for total mitochondrial content (B) or changes in mitochondrial membrane potential (C) by TMRM labeling and flow cytometry. (D) PC3 cells were transfected with control siRNA (Ctrl) or ClpX- or ClpP-directed siRNA, mixed with the ROS scavengers NAC (N) or MitoTempo (MT), alone or in combination, and analyzed by western blotting. Raw data for this figure can be found in S10 Data. (TIF) [file pbio.1002507.s017.tif]

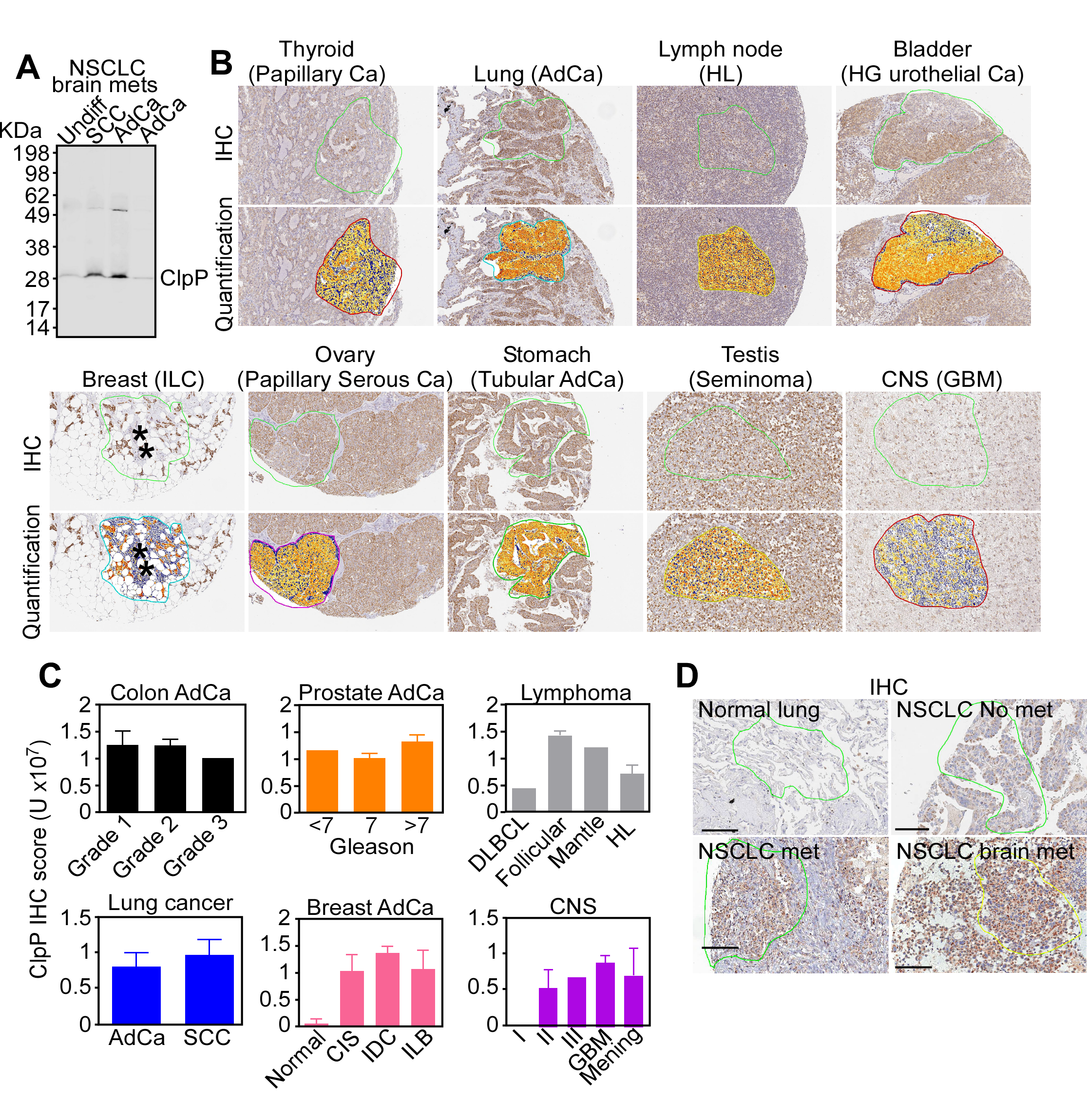

Supplement: S5 Fig — (A) Tissue extracts from brain metastasis of non-small cell lung cancer (NSCLC) were separated by SDS gel electrophoresis and analyzed with an antibody to ClpP by western blotting. Undiff, undifferentiated; SCC, squamous cell carcinoma; AdCa, adenocarcinoma. (B) Primary tissue samples representative of the indicated tumor diagnoses were stained with an antibody to ClpP and analyzed by immunohistochemistry (IHC). Quantification of cytosolic ClpP staining in the marked tissue areas was carried out using the Aperio software (Quantification). Ca, carcinoma, AdCa, adenocarcinoma; HL, Hodgkin’s Lymphoma; HG, high-grade; IDC, infiltrating ductal carcinoma; ILC, infiltrating lobular carcinoma; GBM, glioblastoma. (C) Correlation between ClpP immunohistochemical (IHC) staining in primary human tumors and tumor grade (Colon AdCa, CNS tumors), Gleason score (prostate AdCa), lymphoma subtype (DLBCL, diffuse large B cell lymphoma; Follicular, follicular lymphoma; Mantle, mantle cell lymphoma; HL, Hodgkin’s lymphoma); histotype (lung cancer or breast AdCa; CIS, carcinoma in situ; IDC, infiltrating ductal carcinoma; ILB, infiltrating lobular carcinoma). Mening, meningioma. Data are expressed as mean ± SEM of a ClpP IHC score per each tumor type examined. (D) Primary tissue samples representative of normal lung, non-small cell lung cancer (NSCLC) that developed (met) or not (no met) distant metastases during a 5-years follow-up, or metastatic NSCLC to the brain were analyzed for ClpP expression by immunohistochemistry (IHC), with quantification of marked areas by Aperio. Raw data for this figure can be found in S11 Data. (TIF) [file pbio.1002507.s018.tif]

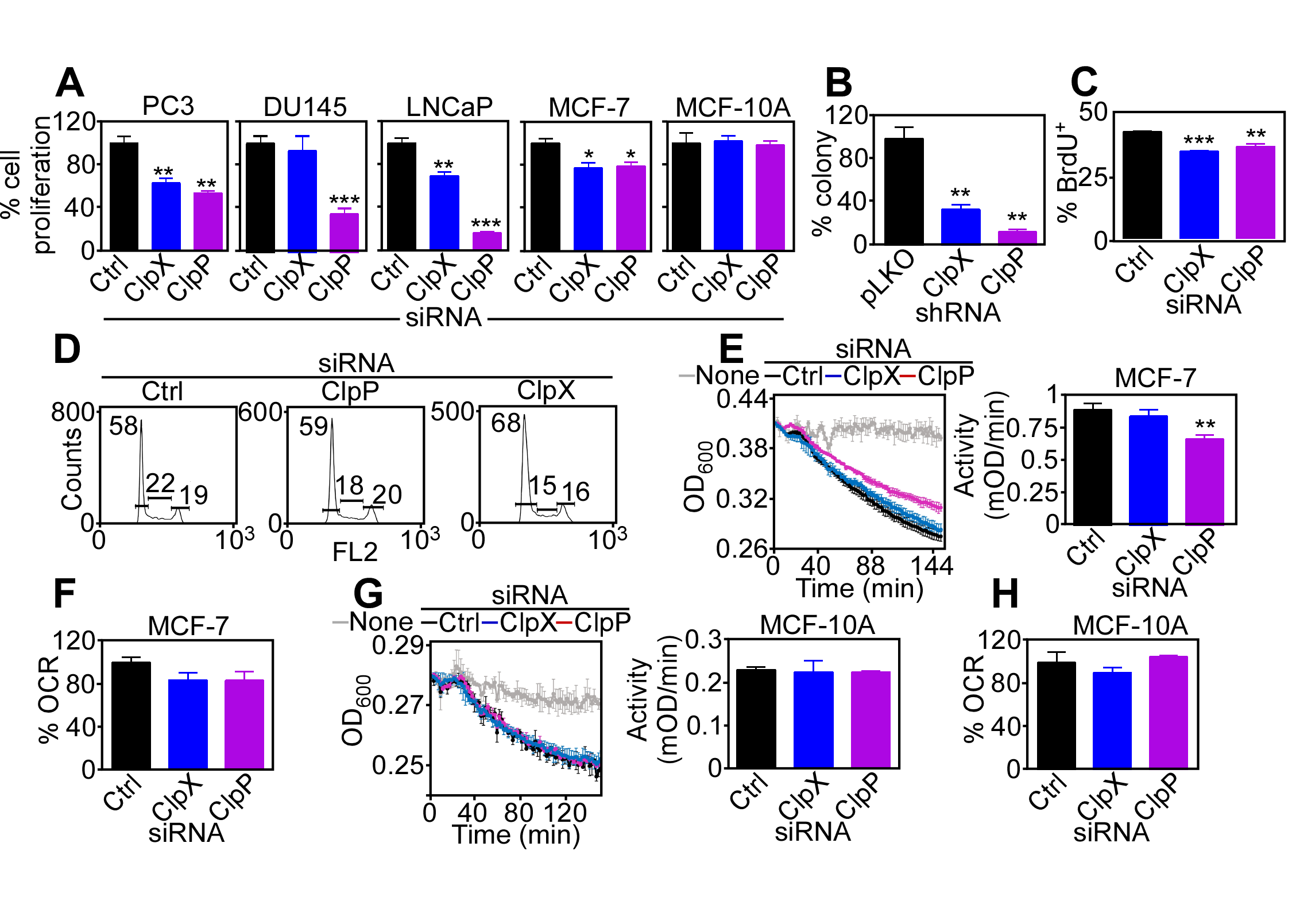

Supplement: S6 Fig — (A) The indicated tumor cell types were transfected with control siRNA (Ctrl) or ClpX- or ClpP-directed siRNA and analyzed for changes in cell proliferation by direct cell counting. *, p = 0.013–0.015; **, p = 0.001–0.007; ***, p < 0.0001. (B) PC3 cells stably transfected with control pLKO or shRNA to ClpX or ClpP were analyzed for colony formation after 7 d by crystal violet staining. **, p = 0.001–0.004. (C) PC3 cells transfected with the indicated siRNAs were analyzed for BrdU incorporation by flow cytometry, and the percentage of BrdU+ cells was quantified. **, p = 0.004; ***, p < 0.0001. (D) PC3 cells transfected as in (A) were analyzed for DNA content by propidium iodide staining and flow cytometry, and the percentage of cells in each cell cycle phase was quantified. (E) Breast adenocarcinoma MCF-7 cells were transfected with control siRNA (Ctrl) or ClpP- or ClpX-directed siRNA and analyzed for Complex II activity. Right, Quantification of citrate synthase-normalized Complex II activity. **, p = 0.002. (F) MCF-7 cells transfected with the indicated siRNAs were analyzed for oxygen consumption rates (OCR). (G) Breast epithelial MCF-10A cells were transfected with control siRNA (Ctrl) or ClpP- or ClpX-directed siRNA and analyzed for Complex II activity. Right, Quantification of citrate synthase-normalized Complex II activity. (H) siRNA-transfected MCF-10A cells as in (G) were analyzed for oxygen consumption rates (OCR). Raw data for this figure can be found in S12 Data. (TIF) [file pbio.1002507.s019.tif]

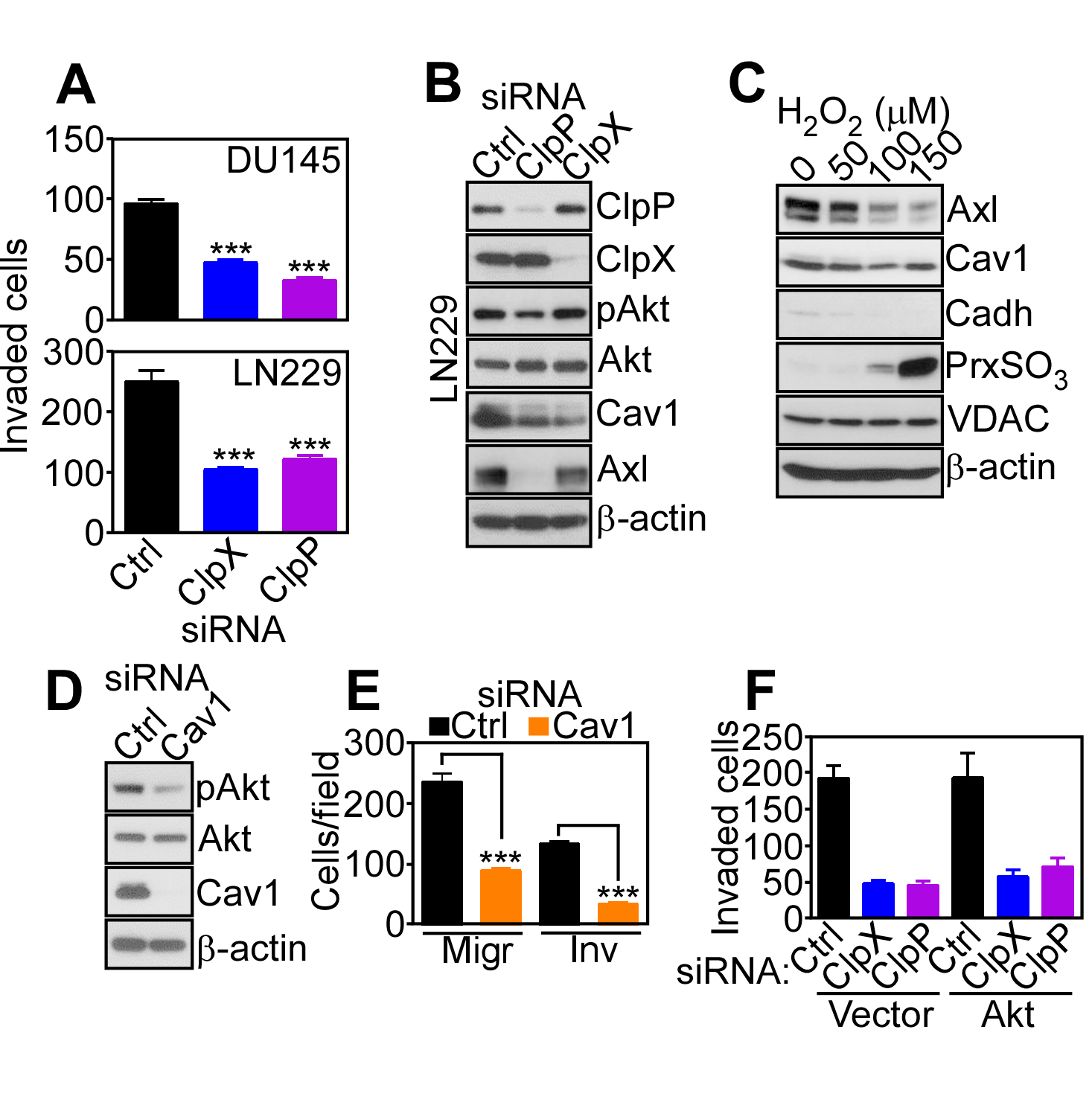

Supplement: S7 Fig — (A) DU145 or LN229 cells were transfected with control siRNAs or ClpX- or ClpP-directed siRNA and analyzed for Matrigel invasion. ***, p < 0.0001. (B) LN229 cells were transfected with the indicated siRNAs as in (A) and analyzed by western blotting. (C) PC3 cells were treated with the indicated increasing concentrations of H2O2 and analyzed by western blotting. (D and E) PC3 cells were transfected with control siRNA (Ctrl) or Caveolin-1 (Cav1)-directed siRNA and analyzed by western blotting (D) or changes in cell migration (Migr) or cell invasion (Inv) across Transwell membranes (E). ***, p < 0.0001. (F) PC3 cells transfected with control siRNA (Ctrl) or ClpX- or ClpP-directed siRNA were reconstituted with vector or Akt cDNA and analyzed for cell invasion in a Transwell assay. Raw data for this figure can be found in S13 Data. (TIF) [file pbio.1002507.s020.tif]
