# Supplementary material for: The Mitochondrial Unfoldase-Peptidase Complex ClpXP Controls Bioenergetics Stress and Metastasis
Source: PLoS Biol. 2016 Jul 7;14(7):e1002507. doi: 10.1371/journal.pbio.1002507 (PMC4936714; doi:10.1371/journal.pbio.1002507)
Supplement: S1 Methods — (DOCX) [file pbio.1002507.s021.docx]

**THE MITOCHONDRIAL UNFOLDASE-PEPTIDASE COMPLEX ClpXP CONTROLS BIOENERGETICS STRESS AND METASTASIS**

Jae Ho Seo, Dayana B. Rivadeneira, M. Cecilia Caino, Young Chan Chae, David W. Speicher, Hsin-Yao Tang, Valentina Vaira, Silvano Bosari, Alessandro Palleschi, Paolo Rampini, Andrew V. Kossenkov, Lucia R. Languino, and Dario C. Altieri

**SUPPORTING INFORMATION**

**SUPPORTING MATERIALS AND METHODS**

**Cells and cell culture**. Human prostate adenocarcinoma PC3, DU145, or LnCaP, glioblastoma, LN229, breast adenocarcinoma MCF-7 or non-tumorigenic breast epithelial MCF-10A cells were obtained from the American Type culture Collection (ATCC) and maintained in culture according to the supplier’s recommendations. Hypoxic treatment was carried out using an enclosed chamber (BioSpherix) flushed with a nitrogen and CO_2_ gas. The O_2_ and CO_2_ concentrations in the chamber were maintained at 0.5%, and 5%, respectively, using a carbon dioxide & oxygen controller (BioSpherix). These conditions were maintained constant throughout the course of the experiments. For galactose challenge experiments, cells were cultured in RPMI 1640 medium without glucose and without sodium pyruvate (GIBCO 11879020) supplemented with 10% dialyzed FBS, 2 mM L-glutamine and 11.6 mM of a mix of D-(+)-galactose and D-(+)-glucose at the indicated proportions. For amino acid starvation experiments, cells were cultured in low amino acid medium (Eagle’s MEM supplemented with 10% dialyzed FBS and 11.6 mM D-(+)-glucose but without sodium pyruvate) for the indicated times. This low amino acid medium contains 50% of the essential and non-essential amino acids included in the RPMI 1640 media formulation.

**Antibodies and reagents**. The following antibodies to ClpP (Cell Signaling), ClpX (Abcam), β-actin (Sigma), VDAC (Cell Signaling), SDHA (Abcam), SDHB (Abcam), SDHC (Abcam), catalase (Cell Signaling), peroxiredoxin-3 (Prx 3, Santa Cruz Biotechnology), 2-Cys Prx-SO_3_ (Thermo Fisher Scientific Inc.), LonP1 (NOVUS Biologicals), Caveolin-1 (Cav1, Cell Signaling), Axl (Cell Signaling), β-catenin (Cell Signaling), cdc42 (Cell Signaling), N-cadherin (Cell Signaling), Ser473-phosphorylated AKT (Cell Signaling), AKT (Cell Signaling), Tyr527-phosphorylated Src (Cell signaling) were used. An oxidative phosphorylation antibody cocktail (Mitoscience) directed against NDUFB8 (subunit of Complex I, 18 kDa), SDHB (subunit of Complex II, 29 kDa), UQCRC2 (subunit of Complex III, 48 kDa), Cox II (subunit of Complex IV, 22 kDa) and ATP5A (subunit of Complex V, 54 kDa) was used. For immunofluorescence experiments, Phalloidin Alexa488, MitoTracker Red CMH2XROS, MitoSOX Red (Molecular Probes) were used. The glucose analog, 2-deoxy-D-glucose (2-DG, Sigma), Complex I small molecule inhibitor, Rotenone (Abcam), and ROS scavengers, *N*-Acetyl Cysteine (NAC, Sigma) or MitoTempo [(2-(2,2,6,6-tetramethylpiperidin-1-oxyl-4-ylamino)-2-oxoethyl)triphenylphosphonium chloride] (Sigma) were used. 2-DG and DCA were used at a concentration of 10 mM, and Rotenone at 1 μM, as described [1].

**Transfections**. For knockdown experiments using small interfering RNA (siRNA), the various tumor cell types were transfected with control, non-targeting siRNA pool (Dharmacon) or siRNA pools directed to ClpP (Origene Technologies) or ClpX (Dharmacon). The various siRNA oligonucleotides were transfected using Lipofectamine RNAiMAX (Invitrogen), and cells were harvested after 48 h or 72 h for subsequent experiments. For transfection of plasmid DNA, tumor cell types were transfected with 1 μg of plasmid cDNA encoding Caveolin-1 (Cav1, Origene) or superoxide dismutase 2 gene (MnSOD, Origene) using Xtremegene (Roche), and further processed after 48 h. In some experiments, siRNA-transfected cells were incubated with ROS scavengers, NAC (1 mM) alone or in combination with MitoTempo (25 μM) after 6 h, and further processed for subsequent experiments. For reconstitution experiments, PC3 cells were transfected with control siRNA or ClpX- or ClpP-directed siRNA and further transfected after 18 h with cDNA encoding Cav1 or control vector. Forty-eight h after plasmid transfection, cells were analyzed for protein knockdown or over-expression and processed for analysis of Matrigel invasion.

**Generation of stable cell lines**. Prostate adenocarcinoma PC3 or DU145 cells were infected with lentiviral particles encoding short hairpin RNA (shRNA) targeting ClpP (TRCN0000046859, GCCCATCCACATGTACATCAA and TRCN0000046860, GCTCAAGAAGCAGCTCTATAA), ClpX (TRCN0000118359, CGAAGATATTGAATCTGTGAT and TRCN0000118360, CCTTTGTATCATCCACCCGTT), or control pLKO plasmid (Sigma). The various trasduced cells were selected in culture medium containing 2 μg/ml puromycin for 10 d, and individual clones from each cell type were established and characterized for stable silencing of ClpP or ClpX, by Western blotting.

**Mitochondria isolation**. Mitochondrial fractions were prepared from PC3 cells using a mitochondria isolation kit (Fisher Scientific), as described previously [1]. Briefly, PC3 cells were mechanically disrupted by 70 strokes in a Dounce homogenizer in isolation buffer A plus aliquots of protease inhibitor cocktail. Cell debris and nuclei were removed by centrifugation at 700 g for 10 min, and mitochondrial fractions were precipitated by centrifugation at 3000 g for 25 min. To obtain highly enriched mitochondrial fractions, samples were subject to another round of centrifugation at 12,000 g for 10 min in isolation buffer C, and the final pellet was used as isolated mitochondrial fractions. To test the impact of ClpXP targeting on mitochondrial inner membrane potential, PC3 cells stably transfected with control pLKO or ClpP- or ClpX-directed shRNA were incubated with 0.1 μM tetramethylrhodamine methyl ester (TMRE), and analyzed for changes in fluorescence emission (FL2) by flow cytometry.

**Protein analysis**. Protein lysates were prepared from the different cell types in RIPA buffer (150 mM NaCl, 1.0% Triton X-100, 0.5% sodium deoxycholate, 0.1% SDS, 50 mM Tris, pH 8.0) in the presence of EDTA-free Protease Inhibitor Cocktail (Roche) and Phosphatase Inhibitor Cocktail (Roche). Equal amounts of protein lysates were separated by SDS gel electrophoresis, transferred to PVDF membranes and incubated with primary antibodies of various specificities. Protein bands were visualized by chemiluminescence. In some experiments, PC3 cells were lysed by sonication in Tris-HCl, pH 7.5, containing protease inhibitors (Roche). For immunoprecipitation experiments, aliquots (500 μg) of total cellular lysates or mitochondrial fractions from control or PC3 transfectants were incubated with non-binding IgG or an antibody to survivin (SVV), ClpP or ClpX for 16 h at 4ºC. Immune complexes were recovered from addition of Protein A-sepharose beads (Calbiochem) for 2 h at 4ºC. After washes in TBST, the immune complexes were separated by SDS gel electrophoresis, and analyzed by Western blotting.

**Gene expression analysis**. PC3 cells were transfected with control siRNA, survivin siRNA or ClpP siRNA for 48h. RNA was extracted using PureLink RNA Mini Kit (Life Technologies). CDNA was generated using TaqMan Reverse Transcription Reagents (Life Technologies). Quantitative PCR amplification reactions were carried out on an ABI 7500 Fast unit. Gene expression levels of Caveolin-1 (Cav1), ClpP and UB32B (control gene) were determined using Power SYBR (Invitrogen). The following primer sequences were used: Cav1 *forward*, CCT TCC TCA GTT CCC TTA AAG C; Cav1 *reverse*,TGT AGA TGT TGC CCT GTT CC; ClpP *forward*, CCT TGT TAT CGC ACA GCT CC; ClpP *reverse*, TGT ACA TGT GGA TGG GCT TC.

**Bioinformatics meta-analysis**. To investigate the prognostic role of ClPp in distant-metastasis formation or cancer recurrence in publicly available patients’ series, we performed a meta-analysis using the web-based interface PrognoScan (<http://www.prognoscan.org/>) [2]. Briefly, we retrieved patients’ outcome data from datasets from which clinical endpoints of disease-free survival, relapse-free survival, event-free survival, progression-free survival, distant metastasis-free survival or distant recurrence-free survival were available. This yielded a total number of 36 datasets (Table S2), which were then filtered for minimum number of patients included in the study (n>50 subjects), absence of pre-surgery therapy (either chemo- or radio-therapies) and with a minimum *p* value (provided within the web-tool) for association of ClpP with patients’ outcome less than 0.05. Fourteen datasets satisfied these criteria, which were next analyzed by sorting patients into “High” and “Low” ClpP-expressing groups using ROC curves and the Youden’s index (J), as described [3]. ClpP prognostic power was assessed by Cox’s regression analysis (Table S2), and Kaplan-Meier survival curves were generated using MedCalc software as described [3].

**Proteomics** **analysis**. Immunoprecipitates were run into an SDS-gel for approximately 5 mm followed by fixing and staining with colloidal Coomassie. The entire region of the gel containing protein was excised and digested with trypsin. Tryptic peptides were analyzed by LC-MS/MS on a Q Exactive mass spectrometer (Thermo Scientific) coupled with a Nano-ACQUITY UPLC system (Waters). Samples were injected onto a UPLC Symmetry trap column (180 μm i.d. x 2 cm packed with 5 μm C18 resin; Waters), and tryptic peptides were separated by RP-HPLC on a BEH C18 nanocapillary analytical column (75 μm i.d. x 25 cm, 1.7 μm particle size; Waters) using a 4 hour gradient. Eluted peptides were analyzed in data dependent mode where the mass spectrometer obtained full MS scans from 400 to 2000 m/z at 70,000 resolution. Full scans were followed by MS/MS scans at 17,500 resolution on the 10 most abundant ions. Peptide match was set as preferred, the exclude isotopes option and charge-state screening were enabled to reject singly and unassigned charged ions. MS/MS spectra were searched using the SEQUEST algorithm in BioWorks (version 3.3.1, Thermo Fisher Scientific) against the indexed human UniRef 100 protein database (September 2013). A list of common contaminants was appended to the human database as well as a decoy database where each protein sequenced was reversed. MS/MS spectra were searched using: partial trypsin specificity with up to two missed cleavages, a 15 ppm precursor mass tolerance, a 20 mmu fragment ion mass tolerance, static modification of cysteine by carbamidomethylation (+57.0215), and variable modifications for both methionine oxidation (+15.9949) and asparagine deamidation (+0.9840). Consensus protein lists were generated by DTASelect v2.0 [4] using the following data filter: 10 ppm precursor mass accuracy, ΔCn ≥ 0.05, full tryptic specificity and requiring a minimum of two peptides per protein [5]. The peptide false discovery rate was less than 1% after data filtering. SVV/IgG protein-fold change was determined from the MS/MS spectra counts calculated on sum of both elutions. To avoid division by zero, proteins not found in the IgG control were assigned a spectra count of 0.5. MitoMiner v3.1 [6] and Gene Ontology annotations (GO:0005739) were used to select only mitochondria-related proteins from the list. Mitochondrial proteins identified with at least 2 MS/MS spectra counts in both elutions of SVV condition and without any MS/MS spectra counts in control IgG samples for both elutions were considered specific survivin-associated proteins. A total of 7 proteins met these criteria.

**Cell proliferation and cell cycle analysis**. Various tumor cell types (2x10^4^ cells) were transfected with control non-targeting siRNA or ClpP- or ClpX-directed siRNA and analyzed for cell proliferation after 24-96 h by direct cell counting. Alternatively, PC3 cells were labeled with 1:1000 dilution bromodeoxyuridine (BrdU) (Amersham Pharmacia Biotech) for 1 h in complete culture medium. Cells were harvested and fixed in cold 70% ethanol followed by incubation with propidium iodide (2.5 µg/ml) in the presence of RNAse A for 10 min at 22ºC, and analyzed by multiparametric flow cytometry with quantification of BrdU^+^ cells and total DNA content using Cell Quest Pro software (Becton Dickinson).

**Colony formation**. PC3 or DU145 cells transfected with siRNA or shRNA to ClpP or ClpX (100 cell per condition) were plated in 6-well plates, and grown in the presence or absence of ROS scavengers NAC (1 mM) and MitoTempo (25 μM) added to the culture every two days. After 7 d, colonies under the various conditions tested were washed with PBS, pH 7.4 and fixed/stained for 30 min in 0.5% w/v crystal violet/methanol. Macroscopically visible colonies were manually counted after rinsing in water.

**Immunohistochemistry**. Sections (4-μm thick) were cut from each tissue microarray (TMA) block and stained with hematoxylin and eosin for morphological analysis or incubated with a rabbit polyclonal antibody to ClpP (1:1000; HPA010649, Sigma Aldrich). Immunohistochemistry (IHC) was carried out with an automatic stainer (Benchmark ULTRA, Ventana), and antibody reactivity was detected with a kit using peroxidase-diaminobenzidine as the chromogen (DAB UltraView, Ventana). All slides were counterstained with hematoxylin. As negative control, one slide was processed without the primary antibody. IHC slides were digitalized using Aperio scanner at 20x magnification, and ClpP cytoplasmic staining in neoplastic epithelial cells was quantified using a specific algorithm implemented in Genie Histology Pattern Recognition software (Aperio, Leica Microsystems). For each tissue core ClpP intensity was calculated using the formula: IHC score (U) = [(n^wp^*I^wp^)+(n^mp^*I^mp^)+(n^sp^*I^sp^)]/ total number of counted cells, were “n” is the number of weakly (wp), moderate (mp) or strong (sp) positive cells and “I” is the staining intensity.

**SUPPORTING REFERENCES**

1. Chae YC, Angelin A, Lisanti S, Kossenkov AV, Speicher KD, Wang H, et al. Landscape of the mitochondrial Hsp90 metabolome in tumours. Nat Commun. 2013;4:2139. Epub 2013/07/12. doi: 10.1038/ncomms3139. PubMed PMID: 23842546; PubMed Central PMCID: PMC3732457.

2. Mizuno H, Kitada K, Nakai K, Sarai A. PrognoScan: a new database for meta-analysis of the prognostic value of genes. BMC Med Genomics. 2009;2:18. doi: 10.1186/1755-8794-2-18. PubMed PMID: 19393097; PubMed Central PMCID: PMCPMC2689870.

3. Vaira V, Roncalli M, Carnaghi C, Faversani A, Maggioni M, Augello C, et al. MicroRNA-425-3p predicts response to sorafenib therapy in patients with hepatocellular carcinoma. Liver Int. 2015;35(3):1077-86. doi: 10.1111/liv.12636. PubMed PMID: 25040368.

4. Tabb DL, McDonald WH, Yates JR, 3rd. DTASelect and Contrast: tools for assembling and comparing protein identifications from shotgun proteomics. J Proteome Res. 2002;1(1):21-6. PubMed PMID: 12643522; PubMed Central PMCID: PMC2811961.

5. Wang H, Tang HY, Tan GC, Speicher DW. Data analysis strategy for maximizing high-confidence protein identifications in complex proteomes such as human tumor secretomes and human serum. J Proteome Res. 2011;10(11):4993-5005. doi: 10.1021/pr200464c. PubMed PMID: 21955121; PubMed Central PMCID: PMC3221390.

6. Smith AC, Robinson AJ. MitoMiner v3.1, an update on the mitochondrial proteomics database. Nucleic Acids Res. 2015. doi: 10.1093/nar/gkv1001. PubMed PMID: 26432830.
