# Supplementary material for: The Mitochondrial Unfoldase-Peptidase Complex ClpXP Controls Bioenergetics Stress and Metastasis
Source: PLoS Biol. 2016 Jul 7;14(7):e1002507. doi: 10.1371/journal.pbio.1002507 (PMC4936714; doi:10.1371/journal.pbio.1002507)
Supplement: S1 Table — NSCLC, non-small cell lung cancer; PNET, pulmonary neuroendocrine tumor; other cancers: breast adenocarcinoma (1), melanoma (1), colorectal adenocarcinoma (1), ovarian carcinoma (1). (DOCX) [file pbio.1002507.s022.docx]

**S1 Table**.

| Disease status | n | Gender  (n, M/F) | Age at surgery  (y, mean with range) | Metastasis at 5 years (n) |
| --- | --- | --- | --- | --- |
| Primary NSCLC | 19 | 15/4 | 67 (57-73) | 9 |
| Brain metastasis from NSCLC | 44 | 29/15 | 63 (38/80) | - |
| Brain metastasis from PNET | 5 | 3/2 | 57 (52/71) | - |
| Brain metastasis from other cancers | 4 | 3/1 | 52 (50/61 | - |
