# Supplementary material for: The Mitochondrial Unfoldase-Peptidase Complex ClpXP Controls Bioenergetics Stress and Metastasis
Source: PLoS Biol. 2016 Jul 7;14(7):e1002507. doi: 10.1371/journal.pbio.1002507 (PMC4936714; doi:10.1371/journal.pbio.1002507)
Supplement: S2 Table — (DOCX) [file pbio.1002507.s023.docx]

S2 Table. Meta-analysis of ClpP prognostic implications.
